# Caring for Carers (C4C): Results from a feasibility randomised controlled trial of positive written disclosure for older adult carers of people with psychosis

**DOI:** 10.1371/journal.pone.0277196

**Published:** 2022-11-08

**Authors:** Christina J. Jones, Cassie M. Hazell, Mark Hayward, Aparajita Pandey, Alexandra Papamichail, Stephen A. Bremner, Daryl B. O’Connor, Vanessa Pinfold, Helen E. Smith

**Affiliations:** 1 School of Psychology, Faculty of Health & Medical Science, University of Surrey, Guildford, United Kingdom; 2 Department of Primary Care & Public Health, Brighton & Sussex Medical School, Brighton, United Kingdom; 3 Sussex Partnership NHS Foundation Trust, Research and Development Department, Sussex Education Centre, Hove, United Kingdom; 4 School of Psychology, University of Sussex, Brighton, United Kingdom; 5 Section of Women’s Mental Health, Department of Health Service and Population Research, IoPPN, King’s College London, London, United Kingdom; 6 School of Psychology, University of Leeds, Leeds, United Kingdom; 7 The McPin Foundation, London, United Kingdom; 8 Family Medicine and Primary Care, Lee Kong Chian School of Medicine, Nanyang Technological University, Singapore, Singapore; PLOS Climate, UNITED KINGDOM

## Abstract

**Background:**

Older adult carers of people who experience psychosis are at increased risk of developing physical and mental health problems due to the compounding factors of supporting their care-recipient and the health changes associated with ageing. Effective interventions exist but can be difficult to access and maintain prolonged engagement. Self-directed writing therapies, frequently referred to as Written Emotional Disclosure (WED), might be a suitable alternative intervention to improve the wellbeing of carers.

**Methods:**

This study aimed to determine the feasibility (recruitment, retention and primary outcome completion) and acceptability of a specific WED intervention known as Positive Written Disclosure (PWD). Informal carers of people with psychosis were randomised to PWD, neutral writing or no writing. Quantitative outcomes including positive and negative affect, carer wellbeing, quality of life, depression, anxiety, stress, self-efficacy, leisure time satisfaction as well as health care utilisation were collected at baseline, 1-, 3-, and 6-month assessments. Qualitative feedback was also collected via questionnaire and semi-structured interviews from those randomised to either writing group.

**Results:**

We successfully met our progression criteria, recruiting to target and within timeframes whilst attaining 97% retention and 84% primary outcome data completed at 6 months. Carers randomised to the positive writing group described the intervention as enabling them to have a more positive attitude and focus on activities for themselves. Both writing groups described their tasks as providing distraction from caring responsibilities. However, some carers found the narrow positive emotion focus challenging.

**Conclusions:**

PWD is a feasible and acceptable intervention for older adult carers of people with psychosis within a community setting. Further refinement of the writing protocol to include choice in type of emotion disclosed in addition to screening for some level of need may be required in future trials to reduce floor/ceiling effects of outcomes which may explain the lack of change observed.

## Background

Of the 6.5 million carers in the UK, 13% provide care for someone with a mental health problem [1]. Over half of all carers (approximately 3.3 million) are aged 50 years or older with the number of carers over 65 years increasing faster than in the general population [1]. In response to a survey of mental health carers, the majority (57%) were found to be over the age of 60 [2]. The carer role is vital both for the person cared for and for society. The costs to the Public Sector associated with schizophrenia alone are estimated at £7.2 billion per year [3]. If informal carers stopped providing care many more vulnerable people would need support from services provided within the Public Sector.

Caring is physically, mentally and emotionally demanding and has been identified as a risk factor for health problems. Up to 40% of carers experience psychological distress and depression; high levels of care provision and age of carer are associated with distress meeting clinical thresholds, as well as a 23% increase in risk of stroke [4–9]. These adverse health effects result from the physical effort required in providing care, and/or dealing with behavioural challenges of the care recipients [4]. In addition there are indirect effects such as less time, energy and finances available for normal daily living. Carers are often mentally and physically exhausted by their caring role and may find it difficult to engage in leisure, social activities or paid employment [10]. Approximately one in five carers give up work to provide care and more than half fall into debt as a result of their carer duties [11, 12].

The psychological and physical health needs of carers of people with psychosis are recognised within the latest National Institute for Health and Care Excellence (NICE) guidance, where mental health services are required to offer carer-specific education and support programmes, as well as access to family therapies [13]. Studies which have explored ways to alleviate stress and improve the wellbeing of carers of people with physical illnesses (such as cancer, heart disease or arthritis) [14] have shown some positive effects of psychosocial therapies, such as community support groups and cognitive behavioural skills training. Specifically, in carers of people with psychosis, psychoeducational interventions were found to improve short-term global morbidity and feelings of burden [15].

Although there are effective interventions to alleviate carer distress, these interventions can be difficult for carers to access and remain engaged with; there are high dropout rates from support groups, with many carers reporting difficulties with finding the time to attend, and others finding it uncomfortable to talk in group settings [16, 17]. In addition, family therapies are rarely appropriate as attending family therapy over a prolonged period is often impractical, especially for those families most in need [18].

Most recently, a self-management toolkit for relatives of people with recent-onset psychosis has been developed and evaluated [19]. During the feasibility study, the use of the toolkit was supported by an early intervention services’ worker offering a face-to-face introductory session followed by one hour each week of telephone or email support for six months. The feasibility study showed improvements in distress, perceived support and perceived ability to cope at six months compared to usual care [19]. However, this intervention targeted relatives who were already receiving support from an NHS early intervention service. To determine clinical and cost effectiveness, the toolkit was adapted for online delivery and included support from trained relatives with lived experience of caring for someone with a severe mental health problem, plus a comprehensive resource directory. This multi-faceted intervention was compared with access to the resource directory alone [20]. Results showed that participants in both groups improved over 24 weeks with no difference between groups. However, inclusion was limited to relatives who were acutely distressed at baseline, so the apparent improvement may be explained by regression to the mean as those with chronic distress were excluded. There remains an urgent need for an easily accessible, effective, self-sustainable, resource-light therapy for carers of patients with psychosis who experience chronic distress due to longer-term caring.

A psychological intervention which has been subject to much empirical research in both clinical and non-clinical populations is Written Emotional Disclosure (WED) [21–37]. This therapy typically involves writing about a stressful or traumatic experience for 20 minutes a day over three consecutive days and has been found to have significant effects on a range of measures of physical and psychological wellbeing [28]. However, limited benefits have been found in carers despite WED being feasible to deliver in a community setting [33–35, 38]. One small study found that changing the focus of the writing to positive events rather than traumatic experiences was associated with greater improvements in psychological wellbeing [39]. This recently developed intervention, referred to as Positive Written Disclosure (PWD), follows an identical format to WED in every other respect.

We know from previous research, that PWD in healthy populations can reduce health complaints and health care utilisation, and improve mood and life satisfaction [40–42]. Most recently, a larger more rigorous three-arm trial of positive, traumatic and control writing tasks in 150 informal carers showed that for some carers, writing about positive experiences led to decreased anxiety and depression for up to 6-months [43]. In contrast, no improvement was found for those carers who undertook traumatic writing. These findings are promising, but the study included carers supporting people of different ages, illnesses or disabilities and may not be generalisable to the carers of people with psychosis. This is because the carers of people with psychosis are longer-term carers who are typically older, are often also providing care to their elderly parents, adult children, and sometimes grandchildren, while are simultaneously managing their own age-related health changes. Their greater emotional strain is compounded further by the increased stigma surrounding psychosis, and poorer satisfaction with support from NHS Mental Health Services due to issues around confidentiality and information-sharing [44, 45]. Therefore, the aims of the Caring for Carers (C4C) trial were to determine whether a definitive trial of PWD in older adult carers of people experiencing psychosis is feasible and justified in terms of recruitment, retention, reasons for drop out and acceptability [46].

## Methods

### Design

This was a feasibility study for a randomised controlled trial (RCT) of carers with three parallel arms (Positive Written Disclosure (PWD), Writing Control (WC), and No Writing (NW)). A Lived Experience Advisory Panel (LEAP), comprising carers of people with psychosis, was convened to support the study including the development of the writing tasks. The sample size justification, predetermined criteria for success and full information on patient and public involvement was prospectively published in our protocol [46]. Ethical approval was granted by the North-West Lancaster Research Ethics Committee (REC reference: 16/NW/0757). This study was registered on 23 January 2017 (Trial registration: ISRCTN79116352).

### Eligibility criteria

Participants were eligible for inclusion if they were fluent in English, aged 50 years or older and identified as a primary carer defined as “a person who provides unpaid support to a partner, child, relative or friend who couldn’t manage to live independently or whose health or wellbeing would deteriorate without this help” [47] to someone with psychosis (including schizophrenia, schizoaffective disorder, schizotypal personality disorder, delusional disorder, psychosis not otherwise specified, bipolar, and depression with psychotic features). The initial protocol stated that eligible carers would be aged 60 years and over however, due to interest in those aged 50 years and over, the inclusion criteria were amended. Carers were excluded if they were currently receiving formal psychological therapy as an individual or family.

### Recruitment

Participants were recruited from NHS (four NHS Trusts in the South East of England) and third sector organisations. Promotional materials were disseminated to potential participants via General Practice carer registers and community mental health teams. Third sector organisations were asked to share details of the study amongst their members. Interested carers responded directly to the research team.

### Randomisation and blinding

Randomisation, conducted by an independent statistician, was to a 1:1:1 ratio using a sequence which was concealed from the research team. The allocation was concealed from participants until they completed their baseline assessment, after which they received an opaque, sealed envelope that contained their allocation. The research assistants remained blinded to allocations throughout the study.

### Intervention and control tasks

Following previous WED protocols [21], participants randomised to PWD were asked to write about a positive and happy experience for 20 minutes on three consecutive days (S1 File). In previous trials of WED, WC participants have been asked to objectively describe images of outdoor scenes [38, 43]. However, the LEAP felt strongly that these images were not neutral in that they may improve mood, given they were aesthetically pleasing, or worsen mood, as carers may be reminded how their outdoor and leisure activities are limited due to caring responsibilities. A set of three images were chosen by the LEAP which depicted rooms within a house, these were considered neutral but with enough detail to write factually and descriptively for the same duration as PWD participants about a different image on three consecutive days. The NW group were given no instructions except to carry on with their usual daily activities.

### Procedure

Participants provided written consent and completed the baseline assessment with a research assistant. Participants randomised to complete a writing task (either PWD or WC) were asked to complete the writing task within a week of completing the baseline assessment. Quantitative outcome data were collected at four-time points; baseline and 1, 3, and 6-months post-baseline. The follow-up assessments were posted to participants with instructions to complete and return in the pre-paid envelope provided. Participants who completed a writing task were asked to return their writing to the research team with their 1-month assessment. After completing the 6-month assessment, participants in the PWD or WC groups were invited to participate in an exit interview.

### Intervention fidelity

To ensure writing instructions were adhered to, we compared the word frequencies between the PWD and WC groups using the Linguistic Inquiry Word Count (LIWC) [48]. Total word count and the number of positive, negative, causation and insight words used by the PWD group was compared with the WC group. We would expect that the PWD participants would demonstrate higher rates on positive, negative, causation and insight words in comparison to the WC participants as these are linguistic markers of emotional expression which are encouraged within emotional writing.

### Measures

#### Feasibility outcomes

1. Completeness of the primary outcome
2. Retention of participants to month 6 follow-up
3. Acceptability of the intervention and trial to participants

Our corresponding progression criteria were (1) at least 80% complete, (2) at least 60% retained, (3) acceptable as assessed in the qualitative interviews.

#### Demographics

Participants were asked their age, sex, employment status, relationship to the care recipient, number of months as a carer, hours per week spent caring, previous experience of therapeutic interventions (e.g. formal family therapy) and history of their chronic medical conditions.

#### Psychological and physical wellbeing

Multiple measures were used to assess wellbeing with psychometric properties published in our protocol [46]:

- Our candidate primary outcome was the Positive and Negative Affect Scale (PANAS) [49]–a 40 item scale with equal number of items measuring positive and negative emotion over the previous week on a 5-point Likert scale, with higher scores indicating greater emotion
- Depression Anxiety and Stress Scale (DASS-21) [50]–a measure of symptoms of depression, anxiety and stress over the past week in both clinical and non-clinical samples. Each scale comprises seven items with higher scores indicating greater impairment
- Carer Wellbeing Scale from the Carer Wellbeing & Support Scale (CWSv2) [51]–a validated tool to measure the experiences of carers of people with mental health problems. This study only used the wellbeing subscale, consisting of 32 items measuring wellbeing in the previous 4 weeks, with higher scores indicating better wellbeing
- General Self-Efficacy Scale (GSES) [52]–a 10 item unidimensional measure of global self-efficacy over the past week with higher scores indicative of greater self-efficacy
- Leisure Time Satisfaction (LTS) [53]–a six item measure developed specifically for carers to capture their engagement in pleasurable activities outside of their caring responsibilities over the past month, with higher scores indicating greater engagement
- EQ-5D-5L [54]–a five item measure and a visual analogue scale designed to capture quality of life cross different domains. An index score, based on the value set for England, was calculated where higher scores indicate better health (scores of 1 represent full health and 0, death) [55]
- General health (mental and physical) was measured by the carer’s use of health services (number of GP visits in the previous month)

#### Experiential feedback

All participants, regardless of which of the three arms they were assigned to, were invited to provide feedback on the study using an end of study questionnaire. Where applicable, participants were asked to respond to statements about study design and writing tasks on a five-point Likert scale, ranging from strongly agree to strongly disagree. In addition, a sub-sample of participants (n = 20) receiving either positive or neutral writing instructions were invited to participate in a face-to-face semi-structured interview to explore their experience (acceptability and feasibility) of the study’s organisation and writing tasks. The interviews were transcribed verbatim and anonymised.

#### Analysis

**Fig 1:**
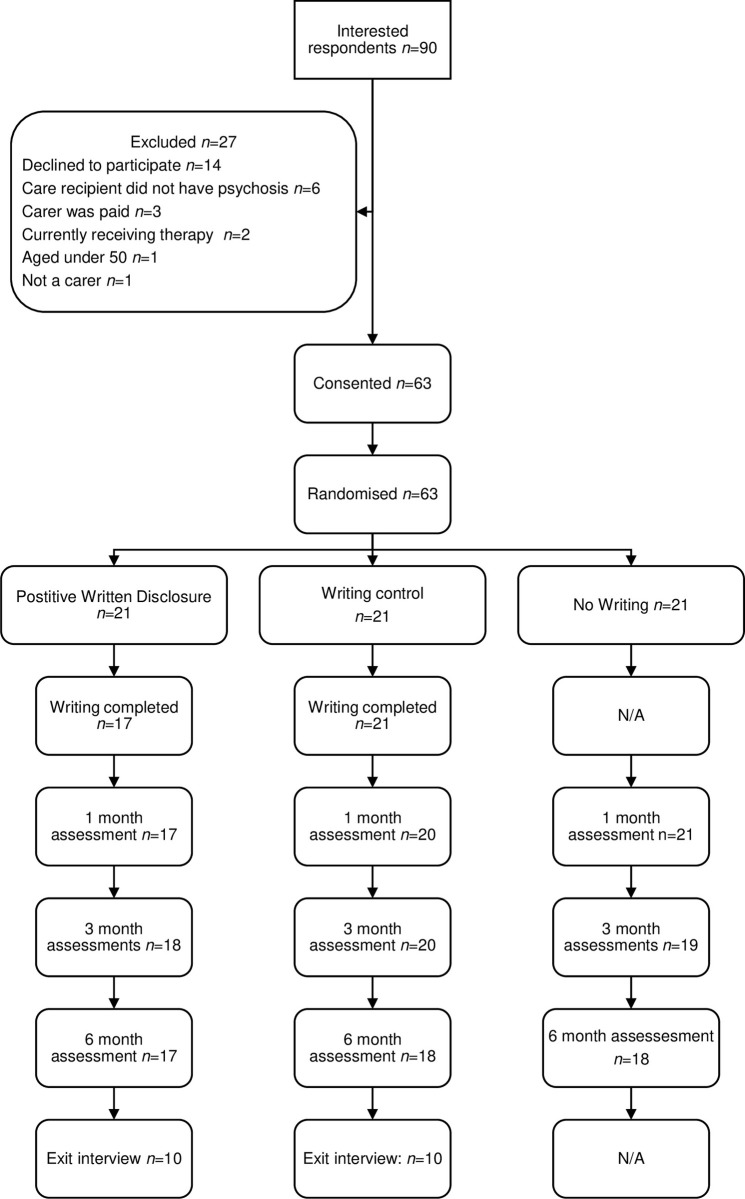
CONSORT flow diagram (Recruitment start date: 09/01/2017, end of follow up: 06/09/2018).

The analysis plan was outlined in our published protocol [46]. For the secondary outcome measures, a random sub-sample of 10% of the participants had their data checked by double data entry against the Case Report Forms by an independent researcher to assess the reliability and quality of the data entry. For the candidate primary outcome, 100% of the data were checked. Flow of participants through the trial is shown in a flow diagram (Fig 1) according to the CONSORT Statement 2010 extension for pilot and feasibility studies [56]. The quantitative data were analysed descriptively, as appropriate for a feasibility study. Means and standard deviations were used to summarise normally distributed variables, medians and interquartile ranges for skewed variables and frequencies and percentages for categorical variables. Effect sizes (Cohen’s d) were calculated to determine a signal of efficacy at 6 months with confidence intervals presented. All quantitative data were managed and analysed using IBM SPSS Statistics for Windows, version 25.0 [57]. As there is no recommended Minimal Clinically Important Difference (MCID) for our primary outcome, the PANAS, a secondary aim was to suggest an MCID, per subscale, for use in future studies as the standard deviation of each PANAS subscale score at baseline, pooled across the three arms, multiplied by 0.2 (denoting a small effect size), as described in a review by Copay et al [58]. The semi-structured interviews with participants randomised to receive the positive and neutral writing were analysed using thematic analysis [59], as was the free text data provided in the feedback form. No software was used in order to manage or conduct the thematic analysis of qualitative data. The LEAP supported the qualitative analysis and assisted in interpretation of the study results as per our protocol.

## Results

### Feasibility

Of the 90 carers who contacted us to enquire about the study, 70% (63/90) were eligible and consented to participate (Fig 1). Almost three quarters of participants were female (73%), and the mean age of the sample was 64 years. Carers were most likely to be parents of the person experiencing psychosis and on average provided care for 61 hours per week and on average, were caring for someone else in addition to the persons with psychosis (Table 1). Of the 42 carers who were randomised to receive writing instructions, 38 (91%) completed and returned all three days of writing. The four participants who did not return the writing exercise were all from the positive writing group. Throughout the 6-month study period only two participants formally withdrew, both citing a relapse in their care recipient’s condition, and giving us a retention rate of 97%. One participant was in the PWD (withdrew after baseline assessment), the second was in the WC group (withdrew prior to the 6-month assessment). At 6-months, primary outcome data (PANAS) was complete for 53 of the 61 remaining carers (87%).

**Table 1:** Demographic characteristics.

	Positive written disclosure (n = 21)	Writing control (n = 21)	No writing (n = 20)*	Overall (N = 62)*
Mean age in years (SD)	64.48 (7.96)	64.10 (7.60)	63.85 (8.64)	64.15 (7.94)
Gender n (%)				
Male	7 (33)	5 (24)	4 (20)	16(26)
Female	14 (67)	16 (76)	16 (80)	46 (74)
Employment status n (%)				
Full time (paid)	-	1 (5)	3 (15)	4 (7)
Part time (paid)	6 (29)	2 (10)	6 (30)	14 (23)
Part time (voluntary)	1 (5)	-	-	1 (2)
Student	1 (5)	-	-	1 (2)
Unemployed (not receiving benefits)	-	2 (10)	1 (5)	3 (5)
Retired	12 (57)	14 (67)	9 (45)	35 (37)
Self-employed	1 (5)	2 (10)	1 (5)	4 (7)
Marital status n (%)				
Single	1 (5)	-	5 (25)	6 (10)
Married/civil partnership	12 (57)	14 (67)	13 (65)	39 (63)
Cohabiting	2 (10)	2 (10)	1 (5)	5 (8)
Separated/divorced	4 (19)	3 (14)	-	7 (11)
Widowed	2 (10)	2 (10)	1 (5)	5 (8)
Ethnicity n (%)				
White British	19 (91)	19 (91)	18 (90)	56 (90)
Asian/Asian British	-	-	1 (5)	1 (2)
Mixed ethnicity	-	-	1 (5)	1 (2)
White Other	2 (10)	1 (5)	-	3 (2)
Rather not disclose	-	1 (5)	-	1 (2)
Mean number of people the carer is providing care for (SD)	1.82 (0.60)	2.13 (0.99)	2.38 (2.13)	2.07 (1.30)
Mean number of years spent care giving (SD)	13.50 (11.32)	12.58 (14.04)	15.13 (11.37)	13.71 (12.17)
Mean hours per week spent care giving (SD)	57.05 (52.63)	63.57 (63.71)	62.30 (64.72)	60.95 (59.60)
Relationship to main care recipient n (%)				
Parent	15 (71)	17 (81)	12 (60)	44 (71)
Spouse	4 (19)	3 (14)	5 (25)	12 (19)
Sibling	1 (5)	1 (5)	2 (10)	4 (7)
Uncle	1 (5)	-	-	1(2)
Friend	-	-	1 (5)	1 (2)
Care recipient diagnoses n (%)				
Schizophrenia	6 (29)	7 (33)	11 (55)	24 (39)
Schizoaffective disorder	3 (14)	5 (24)	2 (10)	10 (16)
Psychosis not otherwise specified	2 (10)	1 (5)	1 (5)	4 (7)
Bipolar	7 (33)	2 (10)	4 (20)	13 (21)
Depression with psychotic features	2 (10)	2 (10)	-	4 (7)
Psychosis	1 (5)	3 (19)	1 (5)	5 (8)
First episode psychosis	-	1 (5)	-	1 (2)
Delusional disorder	-	-	1 (5)	1 (2)
Regularly keeps a diary/writes about emotions n (%)	4 (19)	3 (14)	0 (0)	7 (11)

*Baseline data for one participant in the no writing arm was lost

Descriptive statistics are presented (Table 2). The DASS-21, the only measure with clinical thresholds, indicated that carers were within the subclinical ranges at baseline (all scores between normal to mild impairments). Exploration of the descriptive statistics demonstrated that outcomes remained fairly stable with little change evident between groups and follow-up assessments. Effect sizes for final follow-up data were all small to moderate, with confidence intervals showing no evidence of change between either of the writing groups compared with no writing (Table 2). Estimation of possible MCIDs for both the PANAS positive and negative subscales for use in future trials was a secondary aim of the study which were calculated to be 1.6 and 1.7 respectively.

**Table 2:** Means and standard deviations of outcome measures per group at each time point are presented to enable comparison (median and IQR provided in italics also provided for outcomes which were not normally distributed). Effect sizes (Cohen’s d and 95%CI reported for final follow-up data).

Outcomes	Positive written disclosure					Writing control					No writing			
	Baseline n = 21	1-month n = 17	3-month n = 18	6-month n = 17	Effect size (95%CI) at 6-months against no writing	Baseline n = 21	1-month n = 20	3-month n = 20	6-month n = 18	Effect size (95%CI) at 6-months against no writing	Baseline n = 20	1-month n = 21	3-month n = 19	6-month n = 18
^a^ Positive mood [49]	31.76 (6.88)	30.41 (8.03) *32*.*00 (8*.*75)*	30.50 (8.29)	29.59 (6.78) *30*.*50 (7*.*00)*	-0.24 (-0.90,0.43)	30.93 (9.26)	28.30 (7.97)	28.85 (8.37)	26.61 (8.53) *26*.*00 (14*.*75)*	-0.62 (-1.27,0.07)	33.25 (8.38) *36*.*00 (14*.*50)*	28.43 (6.86)	29.32 (6.57)	31.11 (5.83)
^b^ Negative mood [49]	21.29 (9.49) *17*.*50 (11*.*75)*	20.82 (10.89) *16*.*50 (15*.*75)*	22.89 (10.68)	23.41 (10.60)	0.29 (-0.38,0.95)	23.10 (8.15) *23*.*50 (15*.*00)*	23.40 (9.51)	23.20 (9.07)	21.44 (9.04) *22*.*50 (16*.*50)*	0.10 (-0.56,0.75)	22.00 (7.99)	22.67 (8.06)	21.58 (7.43)	20.56 (9.00) *18*.*00 (17*.*50)*
^c^ Stress [50]	13.14 (7.03)	12.12 (7.19) *12*.*00 (7*.*00)*	14.00 (8.70) *10*.*00 (7*.*50)*	14.94 (7.59) *14*.*00 (5*.*50)*	0.18 (-0.49,0.84)	16.57 (8.22) *16*.*00 (13*.*00)*	15.05 (9.13) *14*.*00 (9*.*00)*	16.99 (8.99)	16.33 (10.39) *14*.*00 (11*.*00)*	0.30 (-0.37,0.95)	13.30 (8.19)	15.90 (7.76) *12*.*00 (13*.*00)*	12.98 (6.66)	13.44 (8.99)
^c^ Anxiety [50]	6.00 (5.44) *4*.*00 (7*.*50)*	5.53 (4.43) *4*.*00 (5*.*00)*	5.89 (6.00) *4*.*00 (6*.*00)*	7.06 (5.34)	0.08 (-0.58,0.74)	8.22 (6.27)	8.48 (7.38) *6*.*00 (8*.*83)*	8.84 (6.98)	10.48 (7.75)	0.52 (-0.15,1.17)	6.14 (5.21)	7.62 (7.14)	5.68 (5.43) *4*.*00 (11*.*00)*	6.52 (7.46) *4*.*00 (7*.*67)*
^c^ Depression [50]	10.10 (8.98) *6*.*00 (11*.*50)*	10.00 (8.51)	9.67 (7.20) *10*.*00 (12*.*00)*	11.41 (5.65)	0.00 (-0.67,0.66)	9.52 (6.48) *8*.*00 (11*.*00)*	11.14 (9.35) *10*.*00 (12*.*00)*	11.46 (9.49) *8*.*00 (14*.*00)*	13.89 (11.11)	0.25 (-0.41,0.90)	8.60 (7.79)	11.22 (9.55)	11.16 (9.55) *10*.*00 (9*.*00)*	11.44 (8.28)
^d^ Carer wellbeing [51]	81.86 (26.90)	92.54 (27.43)	89.62 (27.13) *98*.*50 (55*.*00)*	83.79 (25.84)	-0.18 (-0.85,0.48)	78.90 (25.66)	86.19 (25.85)	84.59 (23.98)	86.28 (28.03)	-0.09 (-0.74,0.56)	80.35 (23.10)	79.94 (24.60)	86.87 (24.52)	88.83 (27.91)
^e^ Self-efficacy [52]	30.31 (4.38)	30.53 (5.00) *30*.*00 (9*.*00)*	29.77 (5.52)	29.31 (4.40)	-0.30 (-0.96,0.37)	29.33 (4.21)	29.50 (4.88) *29*.*00 (4*.*50)*	29.00 (3.45) *29*.*00 (3*.*00)*	29.06 (6.28)	-0.28 (-0.93,0.38)	31.60 (4.69)	29.05 (6.10)	30.89 (4.70)	30.44 (2.96) *30*.*00 (3*.*00)*
^f^ Leisure time [53]	7.14 (3.10)	7.29 (3.13)	6.28 (3.21) *6*.*00 (6*.*00)*	7.00 (2.96)	0.09 (-0.58,0.75)	6.05 (2.60)	5.50 (2.61)	6.56 (3.25)	6.17 (3.47)	-0.14 (-0.79,0.52)	7.35 (3.65)	5.30 (3.10) *6*.*00 (4*.*80)*	5.89 (3.54) *8*.*00 (6*.*50)*	6.69 (3.85) *6*.*00 (7*.*10)*
^g^ Quality of life [54]	0.86 (0.12)	0.86 (0.09) *0*.*87 (0*.*18)*	0.86 (0.08)	0.85 (0.09)	0.34 (-0.34,1.00)	0.81 (0.17) *0*.*84 (0*.*16)*	0.80 (0.19) *0*.*84 (0*.*17)*	0.80 (0.20) *0*.*86 (0*.*23)*	0.78 (0.24) *0*.*83 (0*.*34)*	-0.15 (-0.80,0.50)	0.83 (0.18) *0*.*90 (0*.*18)*	0.83 (0.11) *0*.*86 (0*.*20)*	0.80 (0.17)	0.81 (0.14)
^h^ Self-rated health [54]	71.95 (17.26) *70*.*00 (30*.*00)*	72.24 (17.48)	75.39 (18.72)	70.94 (18.94) *75*.*00 (19*.*00)*	0.03 (-0.64,0.69)	68.29 (16.66) *74*.*50 (20*.*00)*	67.29 (15.87)	68.11 (22.19)	60.41 (23.25) *57*.*50 (43*.*75)*	-0.43 (-1.08,0.24)	74.25 (16.00) *75*.*00 (25*.*00)*	68.67 (19.26) *70*.*00 (22*.*50)*	73.16 (20.76) *80*.*00 (25*.*00)*	70.39 (23.06) *75*.*00 (40*.*00)*
Number of GP visits	0.48 (0.81) *0*.*00 (1*.*00)*	1.06 (2.16) *0*.*00 (1*.*00)*	0.89 (1.08) *1*.*00 (1*.*75)*	1.41 (1.84)	0.08 (-0.58,0.74)	0.62 (0.97) *0*.*00 (1*.*00)*	0.62 (0.86) *0*.*00 (1*.*00)*	1.60 (2.21) *1*.*00 (2*.*50)*	1.94 (2.15) *1*.*00 (2*.*00)*	0.37 (-0.30,1.02)	1.05 (1.57) *1*.*00 (1*.*75)*	0.70 (0.73)	1.26 (2.26) *1*.*00 (1*.*00)*	1.28 (1.32)

^*a*^ Range 10–50 with higher scores indicative of greater levels of positive affect;

^b^ Range 10–50 with lower scores indicative of lower levels of negative affect;

^c^ Range 0–42 with higher scores indicative of greater distress;

^d^ Range 0–128 with higher scores indicative of greater wellbeing;

^e^ Range 10–40 with higher scored indicative of greater feelings of self-efficacy;

^f^ Range 0–12 with higher scores indicative of greater satisfaction in leisure time;

^g^ Range 0–1 with higher scores indicative of better quality of life;

^h^ Range 0–100 with higher scores indicative of better health

### Intervention fidelity

The total word count over the three days between groups were similar, indicating that carers in both groups wrote approximately the same amount. Observations from the Linguistic Inquiry Word Count (LIWC) [48] confirmed adherence to the writing instruction with the number of positive and insight words used by carers in the PWD group was substantially greater than in the WC group. There was only a small increase in use of causation and negative words in the PWD group compared to the WC group (Table 3).

**Table 3:** The characteristics of the writing in the two writing interventions, Positive Written Disclosure (PWC) and neutral writing in the WC group.

Writing category	Positive written disclosure (mean, sd) n = 17	Writing control (mean, sd) n = 21
Total words	1247.12 (454.16)	1066.95 (268.88)
Positive words	12.48 (3.54)	1.93 (0.95)
Negative words	2.36 (1.33)	0.55 (0.43)
Causation words	1.57 (0.82)	0.22 (0.33)
Insight	6.38 (1.90)	1.00 (0.56)

### Participants’ experience

#### End of study questionnaire

Forty participants (66%) completed the optional, anonymised end of study feedback questionnaire (Table 4). The majority of participants were clear on the study processes, how to contact the research team and would recommend other carers to participate. Although still in the minority, the greatest difficulties experienced were the relevance of questionnaires and finding time to complete these (30% and 20% respectively). Only three participants reported disliking the writing task and finding it distressing; with the majority able to fit this into their daily activities.

**Table 4:** End of study participant feedback (n = 40).

Questionnaire item	Proportion agreed or strongly agreed
Participant info sheet easy to understand	98%
Felt able to ask research team questions about study	90%
Understood the randomisation process	95%
Did not like being randomised	18%
Knew how to contact research team	93%
Communication with research team was positive	100%
Understood consent and right to withdraw	100%
Did not mind completing questionnaires	97%
Some of the questionnaires did not seem relevant	30%
Difficult to find the time to complete questionnaires	20%
Writing instructions were clear	97%
Was able to fit the writing takes into my daily activities	86%
Disliked the writing task	10%
Found the writing task distressing	11%
Recommend to other carers of people with psychosis	72%
Would consider taking part in research again in the future	93%

#### Qualitative interviews

The LEAP assisted with identification of the themes generated from interviews with both positive writing participants (n = 10) and participants who received neutral writing instructions (n = 10). There were three overarching themes (changes to thoughts, feelings and behaviours, difficulties encountered and suggestions for broadening the intervention) and eight subthemes, italicised in the subheadings below with participant allocation and identification numbers in parenthesis. Whilst analysis was conducted separately for the two groups of participants, there was significant overlap, as can be seen from the quotations provided.

#### Changes to thoughts, feelings and behaviours

Participants in both groups described benefits of the writing, including its role in *fostering feelings of positivity*, which was unanticipated, particularly amongst the WC group. PWD participants described the writing encouraging them to be more thoughtful, compassionate and resilient and generally considered the writing as therapeutic and uplifting. WC participants on the other hand, reflected more upon the reassurance provided by their needs being the focus of a research project, and how this was sometimes of more benefit than the writing instructions they received.

“*It helped me to get a bit more proactive*… *The process allowed me to be more thoughtful” (PWD participant 001)*

*“I did find it to be quite therapeutic really*… *was able to keep a positive attitude” (PWD participant 023)*

*“It was rewarding cause it was limited only to positive things in one’s past life and it made one go back through one*’*s life searching for these positive things” (PWD participant 031)*

*“It*’*s quite nice to know that carers were being taken notice of” (WC participant 011)*

*“It’s probably being a part of the project in some way that I found beneficial*, *really irrespective of which group I was put in to…And not feeling invisible” (WC participant 059)*

That said, both groups spoke about how the writing instructions gave permission for them to *take time for oneself*. Some participants in the PWD group indicated that they enjoyed the reflective process and that they intended to continue writing after the trial and explore expressive writing. WC participants spoke at length about how the instructions were absorbing and forced them to focus on something for themselves rather than daily chores, giving them a sense of achievement and allowing them to pause and refresh. Given the benefit they experienced, one carer spoke of the need to continue the practice of taking time out during each day for themselves.

“*I am thinking because of this therapeutic value of this study of maybe trying to join a writers’ group or something*. *The moment you put it out and get down to it*, *I found that I really did enjoy it*. *And it was like my time rather than being a carer” (PWD participant 001)*

*“Just being involved and reliving the experience was*, *you know*, *a positive thing*, *it’s been lifting spirits” (PWD participant 045)*

*“It was a bit of time for me to sit down and think about whatever writing I was doing*” *(WC participant 011)*

*“It was just a task that literally took you away from everything that you’d normally be doing and you know*, *allowed you that time to just look at something and then write about it” (WC participant 042)*

*“You are making the effort for the*, *well*, *for yourself in a way” (WC participant 054)*

In addition to taking time for oneself, participants in both groups also spoke about how writing *provided a distraction/escapism*. This again was more commonly reported in WC participants who spoke of the task blocking other thoughts and worries. One carer spoke about the calmness of the images, which also left them feeling relaxed.

“*I was quite engrossed in the writing*, *I wasn’t too aware of my consciousness outside of it” (PWD participant 031)*

*“I do feel I am getting down or if things getting on top of me this is a way of escaping and you know*, *um getting my mind on other things” (PWD participant 045)*

*“I actually did enjoy doing it and it made me feel good*… *It actually did take me away from the situation for that short time” (WC participant 027)*

*“It was something completely different*, *a complete break from you know*, *the routine*, *from day-to-day stuff it was like escapism in a way” (WC participant 042)*

*“It sort of takes you*, *takes you away from*, *from your sort of cares a little bit*, *so it feels like*, *the pictures felt a little bit like a window on to something*, *quite calm pictures” (WC participant 054)*

#### Difficulties encountered

Despite the positive comments articulated, there were a number of difficulties which participants reported. The *narrow focus of intervention and outcome measures* were commented on by both writing groups, although PWD participants tended to describe difficulties in the intervention, specifically in recalling positive experiences or expressing their feelings, leading to feelings of frustration. Whilst WC participants reflected on the fact that the outcomes measured did not allow for full description of their situation or circumstances.

“*I think definitely a degree of frustration “oh there’s got to be something happy that you can think about that happened after you were 16 years old”*, *and that was frustrating because there wasn’t” (PWD participant 050)*

*“My answers to the questionnaires*, *particularly in relation to how optimistic or helpless I felt*, *were completely dominated by the progress of my wife’s illness” (PWD participant 023)*

*“The information that you are getting from me might have been a bit confusing because of all these other things going on*” *(WC participant 022)*

*“There wasn’t*…*anywhere that you could actually say how things had changed cause even though as I said that things had changed but I think it needed to have more*, *you know*, *you was able to write more about the situation” (WC participant 027)*

Only the PWD participants described how *personality factors* might play into the difficulties they encountered with some reflecting on the fact that they found the writing intrusive and expressed a preference to describe factual accounts.

*“Having to draw on emotions*, *was very difficult because I am a very private person and I don’t generally*…*express myself in that way” (PWD participant 005)*

*“It’s always more difficult to write about your own feelings than if its writing fiction*… *so it was quite hard” (PWD participant 008)*

Participants in both groups described *finding the space and time* to complete the writing a challenge due to competing priorities and distractions which is unsurprising given the number of hours the participants in this study typically spent caring.

*“I had to make an effort to find the time so that’s the most demanding part of it*, *you know*, *just making time” (PWD participant 045)*

*“I got lots of responsibilities so*… *finding even 20 minutes is sometimes quite hard” (WC participant 022)*

*“It was just a case of finding*, *if you like*, *some peace and quiet*, *to actually sit down and actually do that without too many distractions” (WC participant 049)*

#### Suggestions for improvement

Both groups provided suggestions, such as the *provision of choice*, which might improve the participant experience. Some PWD participants suggested allowing carers to disclose any emotion, for any time, any duration and using any medium (e.g., typed rather than written) which suited them. Those allocated to the WC groups also reflected on their preference to express emotions rather describe pictures. The PWD participants provided us with useful suggestions regarding the *provision of prompts* which may prove helpful in a definitive trial. These included suggestions of events or experiences (e.g. holidays, birthdays, adventures) as well as ways to structure the writing (e.g. how did you feel before and after?).

## Discussion

This feasibility trial, having met the progression criteria, confirms that a definitive trial of PWD is justified in terms of recruitment, retention, adherence to intervention instructions and overall acceptability. We successfully recruited our target population of carers within the study time frame and retained 97% over the 6-month period of data collection, with complete primary outcome data for 53 carers (87%). The similarity of word frequencies for both writing groups (PWD and WC) together with the increased number of emotive words used in the PWD group is suggestive of adherence to the writing instructions. The carers who participated in the study were largely positive about the organisational aspects of the study and valued the opportunity to participate in research focussed on carers. The majority of participants’ feedback via questionnaires indicated that they were able to find time to complete the questionnaires and incorporate writing tasks into their daily activities. However, several carers, allocated to either writing groups, expressed difficulties during the interviews in finding time to write for 20 minutes, yet also found benefit from having this time for themselves. Those in both writing groups described the intervention as enabling them to have a more positive attitude and provided escapism/distraction from their caring responsibilities.

Our findings of a brief, self-directed intervention for older adult carers appears to have a higher rate of uptake to that of supported self-management interventions for familial carers of people with recent-onset psychosis (19–20%) and other intermediate-intensity family interventions (24–53%) [17, 60]. Our high recruitment and retention rate in a population of long-term carers (with a mean of 13 years of caring experience) may reflect the lack of suitable support available beyond the early episodes of psychosis and established carer support groups such as those run by Rethink Mental Illness. Early Intervention in Psychosis services routinely provide carers’ assessments as well as ongoing monitoring of the needs of the family [19]. In contrast, there are very limited NHS resources available for long term carers whose care recipient is supported by community mental health teams, and so with widespread unmet need a brief intervention was worthwhile and appealing to some. This explanation was supported by the qualitative interviews where participants described feeling *“seen”* by the focus of research.

This feasibility study used rigorous methodology, including independent randomisation and blinded researchers for data collection. A further strength is the creation of our LEAP–a panel of carers who themselves met the study’s eligibility criteria and who advised on the design, recruitment strategies, participant materials and interpretation of qualitative data. Whilst we demonstrated successful feasibility according to our prospectively published protocol [46] and no formal statistical analysis was undertaken in line with guidance for feasibility studies [56], the outcome measures evaluated remained fairly stable throughout the 6-month assessments. Our qualitative data on acceptability was valuable, however the utility of the end of study participant feedback questionnaire was limited by it’s anonymity preventing us from determining differences in satisfaction across the three groups.

The failure to observe change in the outcome measures could be due to a variety of issues.

Firstly, despite the high burden and duration of caring in our sample (which may be synonymous with older adult carers), participants largely scored within the subclinical range for depression, anxiety and stress at baseline. Moreover, our sample reported carer wellbeing at baseline higher than the wellbeing reported at the end of the definitive trial of the REACT toolkit in carers [20]. To mitigate against this in future trials, a screening threshold for caseness will be considered. Secondly, whilst our study intentionally recruited older adult carers of people with psychosis, recent evidence suggests that younger female carers may be in the greatest need of support [61] and that emotional disclosure might prove most beneficial for those who have been caring for less than five years [37]. Future trials should consider stratification on these factors. Finally, prescribing the fixed content of the emotive writing disclosed may be preventing improvements in some carers as evidenced by our qualitative data. A post-study patient and public involvement exercise, with 21 carers of people with psychosis across the UK, facilitated by The McPin Foundation echoed these comments, suggesting that maximum choice of writing topic should be enabled for individuals who have little choice in other aspects of their lives.

We have demonstrated that carers can be successfully recruited and retained within a trial of positive emotional writing. However, the feasibility study has highlighted the need for some refinements to the definitive trial protocol. These include consideration of whether a future trial should be designed to enable participants allocated to emotional writing to select the focus and emotional intensity of their writing. Given the nature of this feasibility study, no definitive clinical implications can be made at this time, but this study does highlight the willingness among long-term carers of people experiencing psychosis to undertake a brief, accessible, unsupported intervention.

## Supporting information

S1 File Positive Written Disclosure instructions. (DOCX)

S1 Checklist CONSORT 2010 checklist of information to include when reporting a pilot or feasibility trial*. (DOCX)

S1 Protocol (PDF)
