# Supplementary material for: Caring for Carers (C4C): Results from a feasibility randomised controlled trial of positive written disclosure for older adult carers of people with psychosis
Source: PLoS One. 2022 Nov 8;17(11):e0277196. doi: 10.1371/journal.pone.0277196 (PMC9642897; doi:10.1371/journal.pone.0277196)
Supplement: S1 File — (DOCX) [file pone.0277196.s001.docx]

Supplementary file 1. Positive Written Disclosure instructions

Day 1

Today I would like you to set aside 20 minutes to write about your very deepest thoughts and feelings about a positive and happy experiences in your life. Think of the most wonderful experience or experiences in your life – happiest moments, ecstatic moments and moments of rapture.

You might tie your topic to your relationships with others, including parents, lovers, friends, or any other people who are important to you. You might link your writing to your future and who you would like to become, to who you were in the past, or to who you are now. Perhaps from being in love or being a parent, from personal achievements at work or in a hobby, or from listening to music or suddenly “being hit” by a book or painting. These are only suggestions however – you may write about any positive and happy experience(s) you like.

Try to imagine yourself in the moment, including all the feelings and emotions associated with the experience – really let go and explore your very deepest emotions and thoughts. It is critical that you really let go and explore your very deepest emotions and thoughts.

Don’t worry about spelling, sentence structure or grammar. The only rule is that once you begin writing, continue to do so until 20 minutes is up. All of your writing will be completely confidential to the study team. We will identify your writing with an ID number rather than your name.

Day 2

Today I would like you to set aside 20 minutes to write about your very deepest thoughts and feelings about a positive and happy experiences in your life. Think of the most wonderful experience or experiences in your life –happiest moments, ecstatic moments and moments of rapture.

You can write about the same thing you wrote about yesterday or you can write about something different, that is entirely up to you. You might tie your topic to your relationships with others, including parents, lovers, friends, or any other people who are important to you. You might link your writing to your future and who you would like to become, to who you were in the past, or to who you are now. Perhaps from being in love or being a parent, from personal achievements at work or in a hobby, or from listening to music or suddenly “being hit” by a book or painting. These are only suggestions however –you may write about any positive and happy experience(s) you like.

Try to imagine yourself in the moment, including all the feelings and emotions associated with the experience –really let go and explore your very deepest emotions and thoughts. It is critical that you really let go and explore your very deepest emotions and thoughts.

Remember there is no need to worry about spelling, sentence structure or grammar. The only rule is that once you begin writing, continue to do so until 20 minutes is up. All of your writing will be completely confidential to the study team. We will identify your writing with an ID number rather than your name.

Day 3

Today I would like you to set aside 20 minutes to write about your very deepest thoughts and feelings about a positive and happy experiences in your life. Think of the most wonderful experience or experiences in your life –happiest moments, ecstatic moments and moments of rapture.

You can write about the same thing you’ve written about on the last 2 days or you can write about something different. The same guidance applies; you might tie your topic to your relationships with others, including parents, lovers, friends, or any other people who are important to you. You might link your writing to your future and who you would like to become, to who you were in the past, or to who you are now. Perhaps from being in love or being a parent, from personal achievements at work or in a hobby, or from listening to music or suddenly “being hit” by a book or painting. These are only suggestions however –you may write about any positive and happy experience(s) you like.

Try to imagine yourself in the moment, including all the feelings and emotions associated with the experience –really let go and explore your very deepest emotions and thoughts. It is critical that you really let go and explore your very deepest emotions and thoughts.

Remember there is no need to worry about spelling, sentence structure or grammar. The only rule is that once you begin writing, continue to do so until 20 minutes is up. All of your writing will be completely confidential to the study team. We will identify your writing with an ID number rather than your name.
