# Supplementary material for: Caring for Carers (C4C): Results from a feasibility randomised controlled trial of positive written disclosure for older adult carers of people with psychosis
Source: PLoS One. 2022 Nov 8;17(11):e0277196. doi: 10.1371/journal.pone.0277196 (PMC9642897; doi:10.1371/journal.pone.0277196)
Supplement: S1 Protocol — (PDF) [file pone.0277196.s003.pdf]

Caring For Carers (C4C)

RESEARCH PROTOCOL
V3: 20.06.2017

Study Title: Caring For Carers (C4C): Pilot randomised control trial of Positive Written Disclosure for Older Adult Carers of people with psychosis.

Study Acronym: C4C

Principal Investigator: Christina Jones [a] C.Jones@bsms.ac.uk

Research Team: Cassie Hazell [a] C.Hazell@bsms.ac.uk
Helen Smith [e] h.e.smith@ntu.edu.sg
Mark Hayward [b] mark.hayward@sussexpartnership.nhs.uk
Daryl O'Connor [c] D.B.O'Connor@leeds.ac.uk
Vanessa Pinfold [d] VanessaPinfold@mcpin.org

Study Sponsor: University of Sussex

[a] *Brighton and Sussex Medical School*
[b] *Sussex Partnership NHS Foundation Trust*
[c] *Leeds University*
[d] *McPin Foundation*
[e] *Lee Kong Chian School of Medicine*

For guidance on filling out the protocol see:
http://www.biomedcentral.com/bmcpublichealth/authors/instructions/studyprotocol

Contents

1 Abstract 3
2 Keywords 3
3 List of abbreviations 3
4 Background 3
4.1 Research question 5
5 Patient and Public Involvement (PPI) 7
5.1 Past PPI 7
5.2 Future PPI 7
6 Methods/Design 7
6.1 Type of study 7
6.2 Participants 8
6.2.1 Inclusion/exclusion criteria 8
6.3 Aims & Objectives 8
6.4 Recruitment and consent methods 9
6.5 Assessment process 12
6.6 Randomisation process & allocation concealment 13
6.7 Procedure 13
6.8 Therapy protocols 15
6.8.1 Positive Written Disclosure (PWD) 15
6.8.2 Writing Control (WC) task 16
6.8.3 Non-Writing Control (NWC) task 16
6.9 Primary & Secondary Outcome Measures 16
6.9.1 Co-Primary Outcomes: 17
6.9.2 Secondary Outcomes: 17
7 Data Management & Analysis 18
7.1 Summary of the Types of Data 18
7.2 Research Variables Form (RVF) 18
7.3 Sample size & Power calculations 19
7.4 Planned data analysis 19
7.5 Dummy results tables 20
7.6 Data collection, entering, coding and checking process 20
7.7 Missing data policy 21
7.8 Potential bias 21
7.9 Data custodian and data ownership 21
7.10 Data quality and Standards 22
7.11 Data security 22
7.12 Data sharing 23
8 Project management 23
9 Ethical considerations 25
10 Discussion of practical and operational issues 26
11 Schedule of events: Project timetable 27
12 Projected outputs and Dissemination 28
13 Plans for Translation 28
14 Gantt Chart 29
15 Appendices 30
16 Amendments 30
17 Competing interests 30
18 Authors' contributions 30

19 Acknowledgements 30
20 References 30

1 Abstract

Background: Older adult caregivers of people experiencing psychosis can experience many physical and mental health difficulties. There is currently little to no provision in healthcare services to meet these needs of caregivers. Due to the demands of caregiving, any intervention would need to be time-limited, and able to fit in around caring activities.

Methods/Design: This study proposes a pilot randomised controlled trial of a self-directed writing therapy called Positive Written Disclosure, compared to a writing control task and a non-writing control group. The study will recruit older adults (aged 50 or over) who currently provide care for someone experiencing psychosis. Positive Written Disclosure will require participants to take 20 minutes out of their day for three consecutive days to write about a positive memory. The writing control task will involve participants writing about a neutral image in a factual and objective way. The non-writing control task will receive no intervention.

Discussion: As this is a pilot randomised controlled trial, the results of this study will help to determine whether a definitive trial is warranted, and if so provide the indices to estimate the relevant parameters required for power calculations.

2 Keywords

Written emotional disclosure, carers, psychosis, older adults, pilot, randomised controlled trial, positive written disclosure.

3 List of abbreviations

WED = Written emotional disclosure
PWD= Positive written disclosure
WC= writing control
NWC= non-writing control
RCT= randomised controlled trial
PIS= participant information sheet
TBC= to be confirmed
CRC= clinical research coordinator
GP= general practitioner

4 Background

Of the 6.4 million caregivers in Britain, one in four cares for someone with a mental illness (Buckner & Yeandle, 2011). In response to a survey on mental health caregivers, the majority (57%) were found to be over 60, with 32% 60-69, 20% 70-79 and 5% over 80 (Pinfold & Corry, 2003). The caregiver role is vital both for the

person cared for and for society as a whole. The costs of caregiving for individuals with schizophrenia alone are estimated at £11.8 billion per year (Knapp, McCrone, Parsonage & Trachtenberg, 2012). If informal caregivers stopped providing this care many more vulnerable people would need the support of NHS services at an estimated total cost of £36,000 per annum per person with schizophrenia (Knapp, McCrone, Parsonage & Trachtenberg, 2012).

Caregiving is physically, mentally and emotionally demanding and has been identified as a risk factor for health problems; up to 40% of caregivers experience psychological distress and depression, and high levels of care provision is associated with a 23% increase in risk of stroke (Haley, Roth, Howard & Sarrod, 2010; Pinquart & Sorenson, 2003). These adverse health effects result from the physical effort required in caregiving, and/or dealing with the behavioural problems of the care recipients (Pinquart & Sorenson, 2003), as well as 'indirect' effects such as less time, energy and finances available for normal daily living. Caregivers are often mentally and physically exhausted by their caregiving role and may find it difficult to engage in leisure/social activities or paid employment (Oyebode, 2003). Approximately one in five caregivers give up work to care and more than half fall into debt as a result of their caregiver duties (Carers UK, 2007; Carers UK, 2008). Indeed, level of caregiver distress has been found to correlate with the number of hours and number of caregiving tasks per week (Hirst, 2005; Martire, Manela, Shuttleworth & Livingstone, 1997).

Surprisingly, very few studies have explored ways to alleviate stress and improve caregivers' psychological and physical wellbeing (Martire, Manela, Shuttleworth & Livingstone, 1997). Psychosocial therapies such as community support groups and cognitive behavioural skills training show some effect on the wellbeing of caregivers of people with physical illnesses, such as cancer, heart disease or arthritis (Martire, Lustig, Schulz, Miller & Helgeson, 2004). Psycho-educational therapies are considered most effective in helping caregivers of individuals with dementia (Alzheimer's disease and others) (Sorenson, Duberstein, Gill & Pinquart, 2006). These therapies typically tend to improve knowledge of the condition, but have only a small effect on cares' psychological wellbeing and depression (Sorenson, Duberstein, Gill & Pinquart, 2006). NICE (2002) recommends that family therapies should be offered to all families of people with schizophrenia for at least 6 months. However, these therapies are difficult to implement, and an audit in East Sussex revealed that family therapies were only deemed appropriate in 42% cases (Beeton, Meddings & Gibbins, 2008). In addition, attending family therapy for a prolonged period is often impractical for those families most in need. Therefore there remains an urgent need for an easily accessible and effective therapy for caregivers of patients with severe mental illness.

A psychological therapy which has received much empirical attention in both clinical and non-clinical populations is written disclosure (see Frattaroli, 2006 for a review). The therapy typically involves writing about a stressful or traumatic experience for 20 minutes a day over three consecutive days and is found to have significant effects on a range of measures of physical and psychological wellbeing. However, very limited benefits have been found in caregivers (Barton & Jackson, 2009; Mackenzie, Wiprzycka, Hasher, Goldstein, & Mackenzie, 2007; Smith et al., 2015). One small study found that caregivers who wrote about positive rather than

traumatic experiences reported greater improvements in psychological wellbeing (Mackenzie, Wipryzcka, Hasher & Goldstein, 2008). Positive written disclosure (PWD) follows an identical format, but participants are asked to write about positive rather than traumatic experiences.

We know from previous research, that positive written disclosure (PWD) in healthy populations reduces health complaints and health care utilisation, and improves mood and life satisfaction (Burton & King, 2004; Burton & King, 2008; Wing, Schutte & Byrne, 2006). Most recently in caregivers, a larger more rigorous three-armed study on 150 informal caregivers trialling positive, traumatic and control writing tasks showed that for some caregivers, writing about positive experiences led to decreased anxiety and depression 2-weeks, 2-months and 6-months later (Ashley, O'Connor & Jones, 2011). Previous studies have reported a strong beneficial effect of positive writing on wellbeing (Burton & King, 2004; Burton & King, 2008; Wing, Schutte & Byrne, 2006). In contrast, no improvement was found for those caregivers who undertook the traumatic writing. Although these findings are promising, this study included a wide range of caregivers, including caregivers of those of any age with physical or mental illness, addiction or disability. These findings cannot be generalised or extrapolated to caregivers of people with psychosis, as these caregivers are generally older and experience greater emotional strain, increased stigma and poorer satisfaction with support given issues around confidentiality and information-sharing (Lindon, 2007).

4.1 *Research question*

This is a pilot study that aims to answer a number of feasibility questions that will determine whether a definitive trial is justified, and if so the design of this trial. Specifically this pilot trial aims to answer the following questions:

a) Are carers of people with psychosis willing to be recruited and randomised?
b) Are there retention issues? If yes, at what stages did these occur? What were the reasons?
c) Are follow-up data complete i.e. no more than 33% attrition?
d) Can the positive written disclosure (PWD) be delivered with sufficient fidelity?
e) Is there sufficient evidence for scaling up to a definitive randomised controlled trial?
f) What sample size is needed to power a full scale randomised controlled trial?
g) Are there any safety issues or adverse events related to the completion of writing tasks?
h) What did the carers think of the intervention and study design?

The research question for the definitive trial will be: Does positive written disclosure (PWD) improve both the psychological and physical wellbeing of older adult carers of

people with psychosis compared to a writing (WC) and non-writing control (NWC) condition?

5 Patient and Public Involvement (PPI)

5.1 Past PPI

Patient and public involvement has been key to the development of this proposal. In one-to-one interviews with three parental caregivers of people with psychosis and one focus group of four attendees of the Carers Centre in Brighton, caregivers identified three needs that writing interventions can address: a) the limited opportunities to express themselves; b) the limitations of support groups to provide peer support because caregivers are already overburdened with their own responsibilities; and c) the limited availability and ability to physically attend meetings. This feedback has been vital in us defining the rationale for this research study.

We also consulted the focus group of carers on the study design, materials, and intervention. Specifically, they helped us consider alternative approaches to recruitment (e.g. third sector organisations), the outcome measures that are meaningful to carers (e.g. wellbeing measures), and most significantly the content of the control conditions (e.g. the image used in the WC group). From these discussions, we added a 'no writing' arm to the trial as carers felt that it might be the act of writing, rather than the content, that is beneficial; they perceived the writing task as offering caregivers an invaluable space where they could focus upon their own needs, rather than the needs of their loved one. All of the study materials were checked by caregivers to ensure that they were clear, accessible, and easily understood.

5.2 Future PPI

A Lived Experience Advisory Panel (LEAP) will continue to be involved in the study until its end. The LEAP will be made of people whom either have previous experience of or are currently a carer for someone with a mental health problem. The group will meet at least three time points during the study with members of the research team to discuss the progress of the study (before ethics submission, after recruitment has started, and after data has been analysed). They will continue to advise on the recruitment and retention procedures (including the participant information sheet), and will consult on the plans for dissemination to participants following data analysis. The LEAP will also assist with the analysis of the qualitative data; once themes are established, they will be invited to look at the themes with supporting excerpts and assess their face validity.

6 Methods/Design

6.1 Type of study

External pilot randomised controlled trial (RCT) with three parallel arms.

6.2 *Participants*

Potential participants will be screened according to the inclusion and exclusion criteria detailed below, using an eligibility assessment (see appendix). All participants will have caring responsibilities for a person with psychosis. Because of the nature of the intervention (PWD), all participants must be able to read, write and communicate in English. This study will only be recruiting carers aged 50 or over.

6.2.1 Inclusion/exclusion criteria

Inclusion Criteria:
All participants must meet the following criteria:

- Classified as a primary caregiver as defined by the Royal College of General Practitioners (2011, p. 9): “any person who provides unpaid support to a partner, child, relative or friend who couldn’t manage to live independently or whose health or wellbeing would deteriorate without this help.”
- Aged 50 years or over
- Providing care for someone with a psychosis diagnosis - psychosis is defined here as including the following diagnoses:
    - Schizophrenia
    - Schizoaffective disorder
    - Schizotypal Personality Disorder
    - Delusional disorder
    - Psychosis not otherwise specified
    - Bipolar
    - Depression with psychotic features
- Able to read, write and communicate in English

Exclusion Criteria:
Participants will be excluded if:

- Currently receiving or have confirmed plans to engage in psychological therapy of any form (including family therapies)

6.3 *Aims & Objectives*

The overall aim of this study is to determine whether PWD improves the wellbeing of older adult caregivers of people with psychosis. The present pilot trial will determine whether the intervention shows promise (determined by between-group effect sizes), and consequently whether a definitive trial is justified. We will use quantitative methods to explore the following aims:

a) To report the means and standard deviations separated by group allocation for all study time points
b) To calculate the group (PWD vs WC vs NWC) x time (baseline vs 1 month/ 3 month/ 6 month) effect sizes and associated 95% confidence intervals for the primary outcome measure
c) To estimate the study recruitment rates
d) To estimate the study retention rates

We plan to use qualitative methods (exit interviews, and anonymous feedback forms) to explore the following aims:

a) The acceptability of the conditions (PWD, WC and NWC)
b) The subjective experience of the conditions
c) The subjective experience of the research study
d) What the next steps for the research programme should be

6.4 Recruitment and consent methods

To recruit participants for this study we will employ a number of recruitment strategies. These recruitment strategies are: (1) through clinical services, (2) the research network, (3) via psychosis patients, (4) collaboration with third sector organisations, (5) promotional materials, (6) GP screening, (7) Sussex Partnership NHS Foundation Trust opt out scheme.

Clinical Services: Mental health clinicians working in the host site (Sussex Partnership NHS Foundation Trust) will be asked to identify any potential participants for this study. We will mostly be asking clinicians whom have clients with psychosis to pass on information about the study to their client's carer. The clinicians will be the first to approach the potential participant regarding this research study, and will be encouraged to pass on the study information to any potential participants. If the potential participant is interested in taking part in this study they can either contact the research team directly themselves, or the clinician can make a referral (once the potential participant has shown an interest).

Research Network: The Research Network is a database of patients, carers, and clinicians whom have given their permission to be contacted about research studies by the Research and Development at Sussex Partnership Trust. All members of the Research Network who match the inclusion criteria for the study will be sent information about the study by the R&D Department. Interested potential participants will be able to contact either the R&D Department or the research team directly and enquire about the study.

Via psychosis patients: Where Sussex Partnership NHS Foundation Trust mental health services with a psychosis diagnosis are involved in research studies and clinics, they will be asked if they have a caregiver. Where patients respond 'yes', they will be given the promotional materials from this study. These patients will be asked to pass on the promotional materials to their caregiver – the promotional materials will only be given to patients that given their permission to be contacted about research studies. The promotional materials will contain information on how the caregiver can find out more about the study and get involved. The research team will not make direct contact with these patients. Contact will only be made by research staff (e.g. Clinical Research Coordinators) or research clinicians who have consent to contact the patients in reference to research studies.

Third Sector Organisations: The study will be advertised through third sector organisations that are likely to support eligible participants i.e. carers charities, and/or mental health charities. Contact will be made with third sector organisations to

promote the study. Where possible members of the research team will attend third sector events and/or meetings to promote the study. If potential participants are then interested in taking part in the study they can either contact the research team directly or a referral can be made by a third sector worker.

Promotional Materials: Study promotional materials (i.e. posters and flyers) will be displayed in appropriate venues across Sussex. Study posters will be displayed in GP surgeries, mental health facilities, and appropriate third sector facilities. All of the promotional material will contain information on how to contact the research team if the potential participant is interested in taking part.

GP screening: General Practices in East and West Sussex will search their caregivers register/database and electronic patient records for caregivers using Read codes. Also the appointed 'carer's carer' within each practice will be asked to identify potential participants. The research team will also seek permission to attend Carer's Support events to promote the study. Identified caregivers will be will be sent information about the study by either the GP or a Clinical Research Coordinator (CRC); or they can self-refer. Interested potential participants will be able to contact either the CRC or the research team directly and enquire about the study.

Opt Out System: Sussex Partnership NHS Foundation Trust have an opt out scheme in place for consent to contact about research. Members of the Research & Development Department will contact patients, who have not opted out of being approached about research, to discuss the study and invite their carer (if eligible) to take part. Only patients who have not opted out of being contacted about research will have identifiable personal information reviewed by members of the Trust's Research & Development Department. They will initially be contacted by members of the Research and Development Department by letter. If the carer of this patient is interested in the study they can either contact a member of the Research and Development Department or contact the research team directly for further information. To clarify, the opt out system will be used to identify those patients with a psychosis diagnosis who have not opted out of being approached about research. The initial letter will make it clear that the research team would like them to pass the study information onto their carer wherever possible.

Irrespective of the method used to recruit participants, the research team will not personally contact any potential participants without their verbal consent to do so. The initial contact with the research team will either be initiated by the potential participant themselves (self-referral), via a clinician referral, third sector referral, or via contact with a CRC. The research team will not access patient or GP records to identify participants. Copies of the PIS will be given to potential referrers to pass onto the potential participants. During the first interaction between the potential participant and the research team, if the potential participant has not already received a copy, they will be given a copy of the PIS. They will be asked to read through the information, discuss the study with friends and/or family, and formulate any questions they may have about the study. After a 'cooling-off' period of 24 hours, the research team will contact the potential participant again to see if they would like to take part in the study.

If the potential participant is still interested in taking part in the study, they will be invited to attend an initial meeting. This initial meeting can take place at the potential participants home or a convenient clinical setting (e.g. GP surgery, mental health building). During this first meeting, a member of the research team will discuss the study with the potential participant and answer any questions they may have. If a participant decides to take part they will be asked to complete an eligibility assessment – this will establish whether the participant meets the study entry criteria. If the participant is eligible and they would still like to take part in the study, then they will be asked to sign two copies of a consent form: one copy will be kept by the research team, and the other copy will be for the participant to keep. For participants who choose not to consent they will exit the study at this point. During this meeting, the participant will be asked to also complete the baseline assessment. The baseline assessment is comprised of multiple questionnaires that will be used as the study outcome measures.

For participants that are not eligible to take part they will be told so immediately following the eligibility assessment. These individuals will be informed why they are not eligible for the study. The reason for their ineligibility will be framed in a positive way so as to minimise disappointment. These individuals will also be offered the opportunity to join the Research Network (if they are not already members). Joining the Research Network will mean they will be sent invitations to participate in other research studies which may be more appropriate, as well as invitations to research and development events. Their referrer (for those that did not self-refer) will also be informed that they are not suitable to take part.

To clarify the recruitment and consent process will proceed as follows:

A referral is made:
Either via a clinician, third sector organisation, GP, CRC, or by the potential participant themselves (in response to promotional material or the Research Network)

The potential participant is given a copy of the PIS:
They will be encouraged to read the PIS, discuss it with any family and/or friends, and formulate any questions they have for the research team

There is a 'cooling off' period of 24 hours where the research team will not contact the potential participant

The research team will contact the potential participant to arrange an initial meeting:
Within the initial meeting the participant will have the chance to ask any questions, and complete (1) an eligibility assessment, (2) consent form, (3) baseline assessment

The referrer (unless a self-referral) will be informed that the participant has agreed to take part in the study

6.5 *Assessment process*

Assessment	Carried out by	What the assessment is for	How is the assessment carried out	At what stage is the assessment carried out
Baseline Assessment	RF or RA	Measure outcomes before randomisation	In person Online or paper based	After consent
1 month assessment	P	Measure outcomes post intervention	Via post Online or paper based	4 weeks post randomisation
3 month follow up assessment	P	Measure outcomes at short term follow up time point	Via post Online or paper based	12 weeks post randomisation
6 month follow up assessment	P	Measure outcomes at long term follow up time point	Via post Online or paper based	24 weeks post randomisation
Exit interview	RF or RA	Generate qualitative data on participants experience of PWD	In person or via telephone Audio recorded	

RF= Research Fellow

RA= Research Assistant
P= participant

The assessment will take place in either the participants own home or in a convenient NHS building (e.g. GP surgery or mental health building). Where assessments are carried out in the participant's home, the lone working policy will be followed (see section 9 – ethical considerations). The assessments at the 1, 3 and 6 month time points will not require any direct contact with the research team. The participants will be send the assessment pack in the post with a covering letter. They will be asked to complete the questionnaires and return then in a pre-post envelope. Participants will also have the option to complete all assessments online (except for the exit interview).

6.6 *Randomisation process & allocation concealment*

At the start of the study, the statistician responsible for randomisation will generate a group allocation sequence that will link participant IDs to either positive written disclosure (PWD), writing control activity (WC) or no writing task (NWC). Block randomisation will be used, and will be carried out by a statistician independent of the research team. The statistician will not have access to any of the personal information of the participants and will randomise using the participant identification numbers. Members of the research team will be blind to the size of the blocks. Randomisation will be carried out using a 1:1:1 ratio. The group allocation will be concealed from participants until after the baseline assessment is completed. The researcher responsible for carrying out the assessments will be blinded to the group allocation.

6.7 *Procedure*

The study procedure is outlined in a CONSORT diagram (see below).

Once participants have been randomised to one of the three conditions: (1) PWD, (2) WC, or (3) NWC; they will be informed of the condition they have been allocated to and the relevant next steps. For participants in the NWC group they will be given an empty writing pack and will be asked to continue with their usual daily activities, and wait to receive the one month assessment pack. For those in the PWD or WC groups, they will receive their writing task and the relevant paperwork for this (see section 6.8 for details of the writing tasks).

There will be no further contact between the research team and the participants until the one month assessment – however participants will have the contact information of the research team in the event they need to make contact. At the one month assessment point, participants will be sent an assessment pack. Participants will be asked to complete some questionnaires and return them in a pre-post envelope within a week of receiving them. If participants were allocated to one of the writing conditions (PWD or WC) then we will also ask them to return their writing tasks. The writing tasks will be read by either CJ or MH to check for any signs of distress or risk. A copy of the writing tasks can be returned to the participants at a later date upon their request.

At both the 3 month and 6 month follow up time points, the procedure will follow that described above for the 1 month assessment. The assessments will be posted to participants to complete, and returned in the pre-post envelope. The only exception is that there will be no writing tasks to be returned. Participants will also have the option to complete the 1, 3 and 6 month assessments over the phone with a member of the research team whom is blind to their group allocation. Phone assessments will only be used where participants have been unable to complete the assessment themselves or return the booklet in the post. For all of the documents that need to be returned to the research team (i.e. assessment booklets and writing tasks), participants will be identified using a participant ID – no identifiable information will be recorded on these documents. For participants in the PWD group and the WC group, they will be offered an exit interview either in person or over the phone. The results of the study will be disseminated to all participants; the PPI consultants will help with this process.

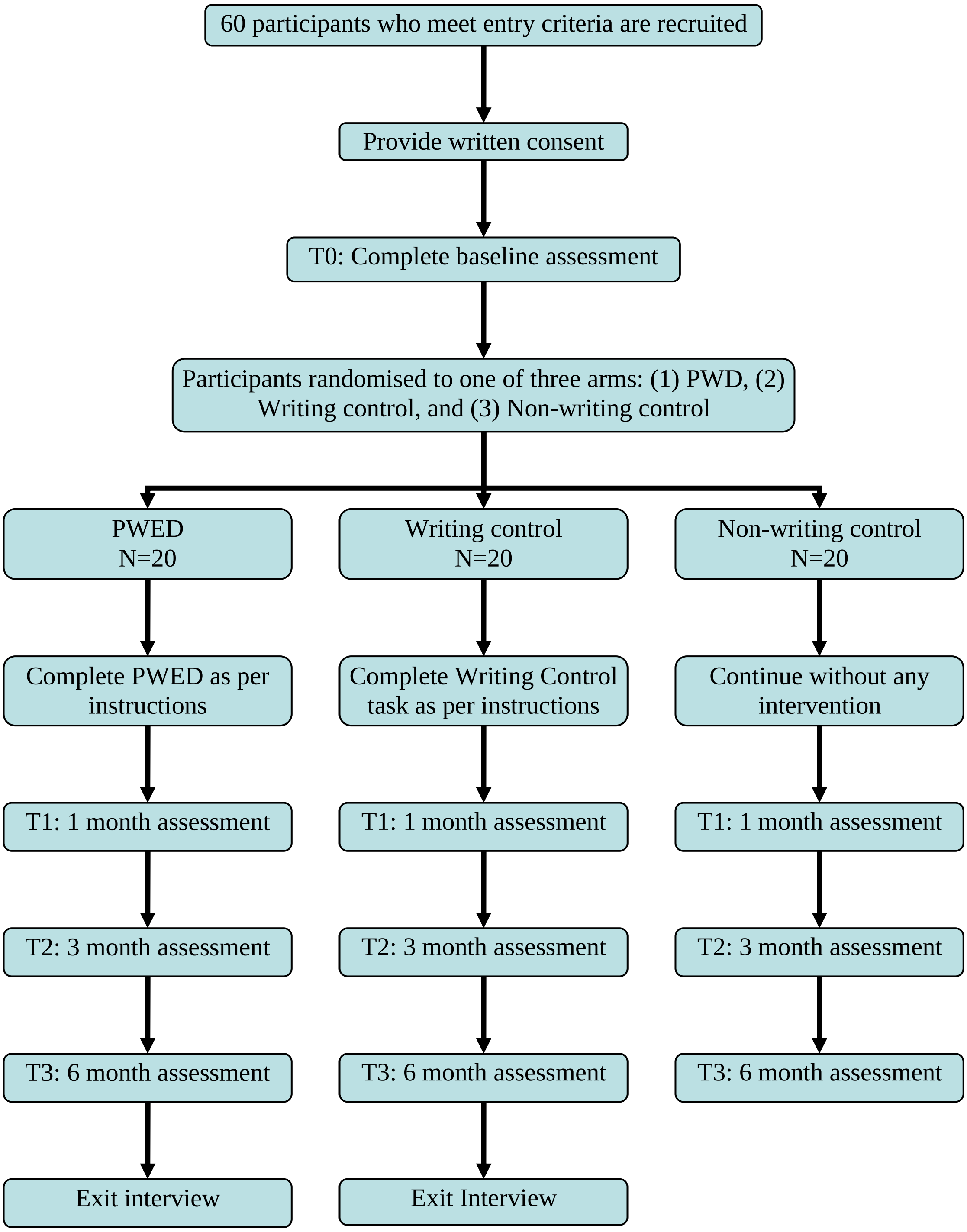

Figure One: Study CONSORT

6.8 Therapy protocols

6.8.1 Positive Written Disclosure (PWD)

The intervention being investigated in the present study is Positive Written Disclosure (PWD). PWD is a self-directed and time-limited therapy. The intervention is based on the wealth of literature on the effects of Written Emotional Disclosure (WED) (Frattaroli, 2006); however in recent years the literature has changes from focussing on negative emotional writing, to writing about positive

emotions (Ashley, O'Connor & Jones, 2011). PWD will require participants to take 20 minutes out of their day, for three consecutive days to write continuously about a positive memory or experience.

Participants will be encouraged to describe this positive event in a lot of detail, focussing on the subjective experience. Participants will be provided with a workbook and a set of writing instructions within which to complete the PWD writing tasks. The PWD workbook and instructions are based on the materials used in similar research studies that were found to be acceptable to participants (Ashley, O'Connor & Jones, 2011; Jones et al., 2015). It is hoped that taking some time to focus on this positive experience or memory will help to improve the wellbeing of our participants (older adult caregivers of people experiencing psychosis).

6.8.2 Writing Control (WC) task

The writing control group within this study will be asked to complete a non-emotive writing task. The participants allocated to this group will also receive a workbook and a set of writing instructions. The writing task will require participants to look at images of different rooms within a house and describe them as accurately and in as much detail as possible. The images were selected to be neutral (not provoke any strong positive or negative feelings) and also rich in detail – so that participants have enough to write about. The final images were selected by our lived experience advisory panel. Participants will be asked to write about these images continuously for 20 minutes over three consecutive days. The instructions for the writing task encourage participants to not give their feelings or opinions towards the images, and instead stick to the facts.

6.8.3 Non-Writing Control (NWC) task

The non-writing control group will not be asked to complete any writing tasks over the course of the study – they will receive no intervention. Participants in this group will be encouraged to carry on with their usual activities, as if they were not part of the research study. So as to maintain researcher blinding, this group will receive a writing pack that is empty. The inclusion of this control group was one outcome of the PPI consultation. Caregivers said that being able to take 20 minutes out of their day to complete a writing task, irrespective of the type of writing, could have some therapeutic effects: caregivers are often preoccupied with the needs of their care recipient, so taking 20 minutes for themselves could provide a brief respite. The non-writing control task will help us to identify whether it is the act of writing or the content of the writing that has beneficial effects.

6.9 Primary & Secondary Outcome Measures

As participants will be require to complete some of the assessments independently (without contact from a member of the research team) all of the measures included are self-report. If participants do have any questions when completing the measures they will be encouraged to contact a member of the research team to discuss this. The measures included in this study have been chosen to capture all of the hypothesised changes that may occur as a result of PWD. The measures

included hope to capture the improvement of positive aspects of the participant's wellbeing, and reductions of the negative aspects.

6.9.1 Co-Primary Outcomes:

The co-primary outcome for this pilot trial will be the Positive and Negative Affect Scale (PANAS) (Watson et al., 1988).
- This measure has two subscales: positive affect, and negative affect
- Each scale is comprised of 20 items
- This measure was chosen because we hypothesise that PWD will increase positive moods and reduce negative ones
- This measure has strong internal consistency (Cronbach's αs> .84)

6.9.2 Secondary Outcomes:

Depression Anxiety and Stress Scale (DASS 21) (Lovibond, 1983).
- This measure has three subscales: anxiety, stress and depression
- Each scale is comprised of 7 items
- This measure will allow us to observe any changes in clinical and sub-clinical levels of anxiety and depression, as well as stress
- This measure has strong internal consistency (Cronbach's α= .88) (Henry & Crawford, 2005)

General Self-Efficacy Scale (GSES) (Schwarzer & Jerusalem, 1995).
- This measure has one subscale: self-efficacy
- The questionnaire is comprised of 10 items
- This measure will allow us to observe any changes in participant's beliefs related to their self-efficacy
- This measure has strong internal consistency (Cronbach's α> .86) (Luszczynska et al., 2005)

Caregiver Wellbeing and Support Scale (CWSv2) (Quirk et al., 2009).
- This measure has one subscale: wellbeing
- Each scale is comprised of 32 items
- This measure will allow us to observe any changes carer-related aspects of wellbeing
- This measure has strong internal consistency (Cronbach's α= .96)

Toronto Alexithymia Scale (TAS 20) (Bagby et al., 1994)
- This measure has three subscales: difficulty describing feelings, difficulty identifying feelings, and externally-orientated thinking
- These scales are made up of 5, 7 and 8 items respectively
- This measure will allow us to identify participants who have difficulty identifying and describing their emotions – this has been previously identified as a moderator of PWD treatment outcomes
- This measure has strong internal consistency (Cronbach's α> .70) (Parker et al., 2003)

EQ-5D health questionnaire (Taylor et al., 1992).
- This measure has one subscale: general health

- This questionnaire is made up of 6 items
- This measure will allow us to observe any changes in participants general health, including both physical and mental health
- This measure has strong internal consistency (Cronbach's α= .85) (Tran et al., 2012)

Leisure Time Satisfaction Measure (LTS) (Stevens et al., 2010).

- This measure has one subscale: satisfaction with leisure time
- This questionnaire is made up of 6 items
- This measure will allow us to observe any changes in participants perceived satisfaction with the quality of their leisure time
- This measure has strong internal consistency (Cronbach's α= .94) (Martinez-Rodriguez et al., 2016)

7 Data Management & Analysis

7.1 *Summary of the Types of Data*

This research study will collect both quantitative and qualitative data. Quantitative data will be collected in the form assessment made up of validated questionnaires. The quantitative data will be collected at four time points: baseline, and 1, 3 and 6 months post randomisation. The questionnaires used have been selected to capture any changes in the hypothesised mechanisms and/or treatment outcomes of PWD. The quantitative data can be completed either using paper-based booklets or online using Bristol Online Survey.

Qualitative data will also be collected in the form of 1:1 interview based on the Change Interview (Elliot et al., 2001). The interview discussion guide was adapted in line with the feedback from the lived experience advisory panel. These interviews will be conducted after all quantitative data has been collected. Exit interviews will only be conducted with those allocated to one of the writing conditions. The interviews will ask participants to talk about their experience of changes during the tasks, if any, their attributions for any changes and their views on the helpful and unhelpful aspects of the intervention.

7.2 *Research Variables Form (RVF)*

The table below details all of the assessments that will be carried out over the course of the study, and at what time point they will be carried out.

Type of data	Variable name	Outcomes/units	Source/Any Instructions
Eligibility assessment	Eligibility Questions	6 items	None
Baseline assessment	Demographics	25 items	None
	DASS 21	21 items	Lovibond, 1983
	PANAS	20 items	Watson et al., 1988
	GSE	10 items	Schwarzer &

			Jerusalem, 1995
	CWSv2	32 items	Quirk et al., 2009
	TAS 20	20 items	Bagby et al., 1994
	EQ-5D	6 items	Taylor et al., 1992
	LTS	6 items	Stevens et al., 2010
1 month assessment	Demographic changes	6 items	None
	DASS 21	21 items	Lovibond, 1983
	PANAS	20 items	Watson et al., 1988
	GSE	10 items	Schwarzer & Jerusalem, 1995
	CWSv2	32 items	Quirk et al., 2009
	EQ-5D	6 items	Taylor et al., 1992
	LTS	6 items	Stevens et al., 2010
3 month assessment	Demographic changes	6 items	None
	DASS 21	21 items	Lovibond, 1983
	PANAS	20 items	Watson et al., 1988
	GSE	10 items	Schwarzer & Jerusalem, 1995
	CWSv2	32 items	Quirk et al., 2009
	EQ-5D	6 items	Taylor et al., 1992
	LTS	6 items	Stevens et al., 2010
6 month assessment	Demographic changes	6 items	None
	DASS 21	21 items	Lovibond, 1983
	PANAS	20 items	Watson et al., 1988
	GSE	10 items	Schwarzer & Jerusalem, 1995
	CWSv2	32 items	Quirk et al., 2009
	EQ-5D	6 items	Taylor et al., 1992
	LTS	6 items	Stevens et al., 2010
Exit interview	Change Interview	N/A	Elliot et al., 2001

7.3 *Sample size & Power calculations*

The trial will recruit 60 participants, with the aim of having 12 completers per condition. A completer is defined as a participant who has completed the baseline and 1 month assessment. This figure is based on the recommendations of Julious (2005) that a pilot RCT should have a minimum of 12 participants per group.

7.4 *Planned data analysis*

For the quantitative data, normally distributed variables will be summarised by their means and standard deviations, skewed continuous variables by their medians and interquartile ranges and categorical variables by their frequencies and percentages. For the co-primary outcome, we will estimate the difference in means,

between the three groups, together with 95% confidence intervals. Available cases will be used, following intention to treat principles.

Qualitative data from the Exit Interview will be analysed using thematic analysis (Braun & Clarke, 2006); this method will highlight the key themes across interviews in relation to the research question. These themes will be used to adapt the intervention where necessary before proceeding to a definitive trial.

7.5 *Dummy results tables*

	Positive Written Disclosure				Writing Control				No Writing Control			
	T0	T1	T2	T3	T0	T1	T2	T3	T0	T1	T2	T3
Measure 1												
Measure 2												
Measure 3												
Measure 4												
Measure 5												
Measure 6												

7.6 *Data collection, entering, coding and checking process*

A member of the research team will conduct the data collection. The data will be managed by a research fellow (CH). All data collected post-randomisation will be collected by a member of the research team whom is blind to the condition the participant has been allocated to or it will be completed without the aid of a researcher (i.e. complete the assessment via post). If an assessor is unblinded at any point, another member of the team who is still blind will be assigned to collect the data wherever possible. If the assessor is unblinded during the data collection, then they will continue with the assessment. The individual conducting the assessment will then be instructed to note that they have been unblinded and this will be reported in the final write up of the study.

The data will be entered into an SPSS file by a research assistant (TBC). The research assistant will be blind to the participant group allocation when entering the data; in other words, the quantitative data entry will be blinded. CH and an independent research statistician will be responsible for analysing the research data. The other members of the research team will supervise the processing of the data. The quantitative data from participants will be entered into the SPSS file as it is collected.

Quantitative data will be collected using either assessment booklets or Bristol Online Surveys (an online questionnaire programme). The questionnaire booklets will be in paper form and will be completed using the relevant instructions for each of the questionnaires. Bristol Online Surveys will be used to collect the same data as the assessment booklets, but will be done online so that the participants' responses can be

immediately downloaded onto SPSS. The method of data collection will vary depending on what is most convenient for the participant.

All members of the research team that are involved in the collection and management of data will be given the necessary training on how to use and administer the clinical measures used in this study. Training will involve a meeting with a member of the team who is experienced in using the particular clinical measure to discuss the questions and instructions. Next, the individual will be required to observe an experienced member of the team using the clinical measure, and then for them to be supervised using the clinical measure. If after this training process there are still problems in their use of the clinical measure, any of the training steps may be repeated until a member of the research who is experienced in using the measure, is confident in the individual’s ability to use the measure.

Once data entry is complete, a 10% random sample of all assessment booklets will be checked against the data entered by a member of the research team whom has not entered any of the data. The two records will be compared to identify any discrepancies.

With regards to the qualitative data, CH and a research assistant (TBC) will be responsible for carrying out 1:1 interviews with participants, and transcribing these. An interview schedule will be used to ensure consistency across the data collection process. At the point of transcription, all identifiable information will be removed. The data will be transcribed into NVivo, and this programme will be used to carry out the qualitative analysis.

7.7 *Missing data policy*

Missing data will be reported but not replaced.

7.8 *Potential bias*

There is potential for bias where those collecting the data are aware of whether the participant is in the intervention or control condition. If the assessor knows the group the participant has been assigned to they may, knowingly or not, have an influence on the responses given by participants. To address this potential for bias, members of the research team will not have direct contact with participants to carry out all post-randomisation assessments: these will be done via post or online. All participants, irrespective of group allocation will be sent the same assessment pack with the same covering letter. In the event that a face-to-face assessment is needed (e.g. participant requests support to complete the assessment), this assessment will be conducted by the study research assistant (TBC) whom will be blinded to the participant’s group allocation. Participants will be asked to not disclose the condition they have been assigned to, to the individual collecting data.

7.9 *Data custodian and data ownership*

Name of data custodian: Miss Cassie Hazell, Brighton and Sussex Medical School, c.hazell@bsms.ac.uk

Name of data owner: University of Sussex.

7.10 Data quality and Standards

The research team adhere to the good practice and standards principles which are set out in the Sussex Partnership Policy for Data Protection, Security and Confidentiality 2013. This policy reflects the recommendations from current legislation, including The Caldicott Report (1997), the British Standard (ISO IEC 27002) for Information Security, the Data Protection Act, 1998 and the Sussex Partnership Foundation Trust Research Policy 2012.

All research will be carried out under the above standards and will be reviewed by an NHS Ethics Committee and given approval by the R&D Department under the NHS Research Governance Framework 2005.

The collecting and analysis of the data will be supervised by researchers within the team: specifically Dr Chrissy Jones, and Dr Mark Hayward. The management of the data will be a regular item at the research team meetings.

7.11 Data security

Participant names will not be used at any point during the collection of data. With regards to the data collection, participants will be identified using a unique number code. A separate file will be kept electronically linking participants names and personal details to the number codes; this file will be kept in a password protected file that only members of the research team will have access to.

The quantitative data collected using paper-based assessment booklets will be kept in a locked filing cabinet at the Brighton and Sussex Medical School. The research fellow (CH) will hold the key for the cabinet. This paper-based data will be kept for two years in line with the Department of Health's guidelines for research management and data storage. Once this time is up it will be destroyed (shredded).

The quantitative data collected using Bristol Online Surveys will be downloaded from the site as soon as it is complete. All of the data collected on BOS will be anonymous, and protected by a BOS password. The data that is downloaded electronically, along with any other electronic data (i.e. the SPSS file with questionnaire scores) will be anonymised, and kept in a password-protected file, on a password-protected computer. Only members of the research team will know the passwords, and will therefore be able to access the electronic data.

The qualitative data will be collected using an audio recording device. Once the interview data has been collected, it will be stored on one computer that is password-protected, within a password-protected file. Only members of the research team will know the password, and therefore have access to the data. Once the data has been downloaded to a computer it will be deleted from the recording device. The

transcripts of the interviews will also be kept in password protected files, that only members of the research team will be able to access. The transcripts will not contain any identifiable information.

The password-protected computers that data will be stored on will be situated at both the Brighton and Sussex Medical School and in the Sussex Partnership NHS Foundation Trust Research and Development Department.

7.12 *Data sharing*

Data may be shared outside of the research team as part of future research studies. Only anonymised data will be shared. This will be make explicit to participants on the study consent form. The participant's personal details will not be shared with anyone outside of the research team. In addition the number of participants recruited, and how they are recruited will need to be recorded and shared with both the Sussex Partnership NHS Foundation Trust research and development department and the National Institute for Health Research. The number of participants recruited will need to be shared to inform recruitment targets for both institutions. None of the personal details of the participants will be shared.

8 Project management

Project Team Member	Role	Contact Details
Christina Jones	Principal Investigator	C.Jones@bsms.ac.uk
Cassie Hazell	Research Fellow and Trial Manager	C.Hazell@bsms.ac.uk
Helen Smith	Researcher	h.e.smith@ntu.edu.sg
Mark Hayward	Researcher	Mark.Hayward@sussexpartnership.nhs.uk
Daryl O'Connor	Researcher	D.B.O'Connor@leeds.ac.uk
Vanessa Pinfold	Researcher	VanessaPinfold@mcpin.org
Stephen Bremner	Statistician	S.Bremner@bsms.ac.uk
TBC	Research Assistant	TBC

Steering Committee	Role/ Responsibilities	Contact Details
Susan Ayers	Chair of TSC with expertise in expressive writing	Sussan.Ayers.1@city.ac.uk
John McCormack	Expertise in psychosis	John.McCormack@scottishrecovery.net
Anna-Marie Jones	Expertise in statistics and research methodology	Anna-Marie.Jones@sussexpartnership.nhs.uk
Kathy Greenwood	Expertise in caregiving	K.E.Greenwood@sussex.ac.uk

Jill Scholl	Expertise in lay involvement	Jill.Scholl@btinternet.com

9 Ethical considerations

Informed consent:

Consent to take part in this study will be informed. All participants will be given the PIS for at least 24 hours before meeting with a member of the research team to discuss consent. Furthermore participants will have the opportunity to ask questions about the research study before signing consent. The combination of the PIS and the chance to ask questions to a member of the research team will mean that any consent given will be fully informed.

Right to withdraw:

All participants will be told both verbally during the consent meeting, and within the PIS that they can withdraw from the research study at any point. Participants will be asked to sign an item on the consent form that confirms their right to withdraw and that they are not obligated to provide a reason for leaving the study.

Confidentiality:

All of the data collected within the research study will be confidential and the information will not be released outside of the research team. The writing tasks that the participants submit will only be identifiable via the participant ID. The members of the team responsible for screening these tasks for signs of risk will not know which participant ID corresponds to which participant. Confidentiality will only be broken if participants disclose any information that would put themselves or another at risk. If information of this nature is shared Sussex Partnership NHS Foundation Trust risk procedures will be followed.

Risk procedures:

If information is shared that presents risk to the participant or someone else, Trust risk procedures will be followed. In the first instance, any issues of risk will be reported to the trial manager (CH). If the issue of risk requires action, this will be taken to the Chief investigator (CJ) and other relevant members of the research team based locally (MH). CH and CJ have experience of running trials with vulnerable populations. MH also has trial management experience, and has previously worked as a clinical psychologist working with people in distress. If the issue cannot be resolved within the research team, the participant’s GP, and any other relevant authorities, will be contacted and made aware of the risk. In emergency situations, the emergency services will be contacted.

Risk to research staff:

There are not believed to be any likely risks to members of the research team in conducting this study. Risk procedures will be put in place in the event of any adverse events. When meeting with participants within NHS buildings, the research team will be instructed to use the available safety devices. For example, within most NHS buildings personal alarms are available that can be used to alert other staff working in the building that someone’s safety has been compromised. Moreover, where referrals are made by third parties, the referrer will be asked to note any risk associated with the participant. Where there are notable risks or violence issues, the research team may choose to decline the referral, or plans can be made to ensure safety for all throughout the research process.

There are likely to be instances where members of the research team visit participants in their homes (for example to complete assessments). When home visits are needed, the Trust's lone working policy will be followed. A home visit will not be made if there are severe risk-related issues connected to the participant. For minor issues of risk, the assessment will be carried out in pairs. Where members of the research team make a home visit, another member of the research team will be made aware of the home visit, including the time and location of the visit – they will expect a phone call from the member of the research at an agreed time to confirm they have left the participant's home. Only the initial meeting (where consent is taken and the baseline assessment is completed) requires face-to-face meetings with the participant.

Anonymity:

The names of participants will not be used in the collection and storing of data. Participants will be assigned a number that will be used in the place of a name. Additionally in the collection of qualitative data, all of the participant's personal details and any other identifiable information will be removed at the point of transcription. Where it is needed, pseudonyms will be used. A separate file, that will be password protected, will detail all of the participants' personal information (e.g. names, addresses, phone numbers) and the number/pseudonym they have been assigned.

Potential for distress:

It is unlikely that participating in this study will cause any distress to participants. Positive Written Disclosure is an intervention that encourages the recipient to focus on position emotions, thoughts and memories – its intention is to improve mood, rather than cause any distress. Similarly the writing control task has been designed to be neutral, and not provoke any emotions (neither positive nor negative).

In over 30 years of research into the effects of writing interventions it is very rare that any participant has become seriously distressed. Trials of Positive Written Disclosure (Ashley et al., 2011) and our trial of negative Written Emotional Disclosure (Jones et al., 2015) have been carried out with caregivers without any adverse outcomes. At the start of the study all participants will be given information about local NHS services (e.g. mental health line), and third sector organisations (e.g. carers charities and support groups) that can provide support in the event that they feel distressed - whether that distress be related to the study or not. We will inform the participant's GP they are taking part in the study, and that if participants show signs of distress we will advise them to see their GP.

10 Discussion of practical and operational issues

Adverse events: The Good Clinical Practice guidelines will be followed in the event of any adverse events during the study. The procedure that will followed is outlined here:

1. If any adverse event occurs this will be taken firstly to the trial manager (CH)
2. The adverse event will then be rated on a 5 point scale for severity and a separate 5 point scale for relevance to the study
3. If the event is deemed to be severe, then this will be taken to the Chief Investigator (CJ) and the wider research team (MH, HS, DO, VP)

4. If there is a cause for concern (i.e. an adverse event was rated as both severe and relevant to the study) this would then be taken to the Trial Steering Committee and the sponsor (the University of Sussex)
5. Both parties will then make a decision as to whether the adverse event needs further investigation, and whether the study needs to be stopped
6. The outcome of any investigation will then determine the future of the study (e.g. whether the study is able to resume again, and if so, when it can resume)

Any severe and relevant adverse events that occur during the research will also be reported to the ethics committee, in line with NHS ethics protocol. Additionally, the participants GP will be informed about the adverse event. Emergency services will also be contacted where needed.

Both the trial manager (CH) and the principal investigator will take joint responsibility for informing both the Trial Steering Committee and the sponsor of any adverse events. Both parties will be informed via email – all emails related to this will be marked as urgent.

Managing risk: In the event there are risk-related issues brought to the attention of the research team (i.e. when a clinical makes a study referral), the study protocol will be adapted to manage these in a safe way. Where the risk is too high and involvement in the research study may put the participant at any further risk, they will not be recruited to participate.

11 Schedule of events: Project timetable

Week?	Event	Who?
0	Promote the study using the various recruitment methods	RF<br>RA
0	Potential participant receives PIS via referrer	
0	Research team receives the referral	
1	First contact is made with participant – the participant is given a copy of the PIS (if they have not received one already)	RF
1	Initial meeting: participants ask questions, eligibility assessment, consent and baseline assessment	RF<br>RA
2	Randomisation to one of the three conditions (PWD, WC or NWC)	S
3	Covering letter and writing task (if required) are send to participants, depending on group allocation	RF
6	1 month assessment: participants are sent the assessment pack to complete, and those whom were allocated to the writing task conditions (WC or PWD) will be asked to return writing tasks	RF
14	3 month assessment: participants are sent the assessment pack to complete	RF
26	6 month assessment: participants are sent the assessment pack to complete	RF
27	Exit interviews will be offered to all participants in the PWD condition	RF<br>RA

RF= Research Fellow

RA= Research Assistant
S= statistician

12 Projected outputs and Dissemination

We plan to disseminate the findings of this study to both academic and non-academic communities. We plan to disseminate the findings in a number of ways: (1) research participants, (2) NHS and clinical community, (3) wider community, and (4) academic community.

Research participants: All participants taking part in the study will receive a summary of the research findings once data have been analysed.

NHS and clinical community: We will arrange for presentations to clinical management groups within Sussex Partnership NHS Foundation Trust and publish summaries of findings within their "Research Magazine" and through their "Research Network". Dissemination nationally will be augmented through our links with Rethink, FACTOR and McPin (when the project is adopted into a CLRN portfolio). We plan to present our findings in caregiver organisation publications (e.g. "Your Voice" by Rethink).

Wider community: Research findings will also be presented at caregivers' groups (e.g. The Caregivers Centre) with the support of the caregiver representatives and a summary included in caregivers' newsletters.

Academic community: Study results will be shared through high impact, academic peer-reviewed journals, using open access journals where possible (e.g. BMJ Open Access). Investigators have a good track record of publication in high impact mental health, psychology and general practice journals. Results will be presented at internal and external seminars. If PWD therapy is found to be feasible and acceptable to caregivers of people with psychosis, a grant to fund a fully scaled RCT to determine therapy effectiveness will be developed and submitted to an appropriate funder (e.g. NIHR).

13 Plans for Translation

As this is a pilot trial there are unlikely to be any immediate changes to health care provision. However this study will be the start of an evidence base that acknowledges the mental and physical health needs of older adult carers of people with psychosis, and seeks to improve wellbeing through a low-cost, time-limited, self-directed intervention. If the findings demonstrate the intervention to be effective, then this will most hopefully lead to a larger scale trial. A larger, fully powered trial will help to provide an evidence base that will be needed for this intervention to be translated into practice.

14 Gantt Chart

	2016							2017												2018				
	June	July	Aug	Sept	Oct	Nov	Dec	Jan	Feb	Mar	Apr	May	June	July	Aug	Sept	Oct	Nov	Dec	Jan	Feb	Mar	Apr	May
Produce protocol, IRAS application and study materials	■	■	■	■																				
Consultation with PPI group				■																				
Edit all materials in light of PPI feedback				■	■																			
Apply for REC approval					■																			
REC approval and portfolio adoption process					■	■																		
RA appointment									■	■	■	■	■	■	■	■	■	■	■					
Brighton GP recruitment							■	■																
Other GP recruitment									■	■	■													
Begin recruitment							■	■	■	■	■	■	■	■	■	■	■	■	■					
Begin randomisation							■	■	■	■	■	■	■	■	■	■	■	■	■					
Begin blinded assessments							■	■	■	■	■	■	■	■	■	■	■	■	■					
Begin interviews								■	■	■	■	■	■	■	■	■	■	■	■					
Data analysis																				■	■	■		
Write up report																				■	■	■	■	
Dissemination																							■	■

Guidance can be found at http://www.biomedcentral.com/bmcpublichealth/authors/instructions/studyprotocol

15 Appendices

All study materials will be included as appendices at the end of this document.

16 Amendments

No amendments to note.

17 Competing interests

There are no competing interests.

18 Authors' contributions

This protocol was written by CH, and edited by CJ and MH. All members of the research team have approved the final version of the protocol. The LEAP have also reviewed the protocol and provided feedback.

19 Acknowledgements

The research team would like to acknowledge all of the people involved in the PPI groups that helped to shape this study; and to Ruth Chandler for coordinating this.

20 References

Andrews, A., Knapp, M., McCrone, P., Parsonage, M., & Trachtenberg, M. (2012). *Effective interventions in schizophrenia the economic case: A report prepared for the Schizophrenia Commission*. London: Rethink Mental Illness.

Ashley, L., O'Connor, D. B., & Jones, F. (2011). Effects of emotional disclosure in caregivers: Moderating role of alexithymia. *Stress & Health, 27,* 376-387.

Barton, K., & Jackson, C. (2008). Reducing symptoms of trauma among carers of people with psychosis: Pilot study examining the impact of writing about caregiving experiences. *The Australian and New Zealand Journal of Psychiatry, 42,* 693-701.

Beeton, L., Meddings, S., & Gibbins, J. (2008). *NICE guidelines schizophrenia report.* Sussex Partnership Trust Internal Report.

Bjelland, I., Dahl, A. A., Haug, T. T., & Neckelmann, D. (2002). The validity of the Hospital Anxiety and Depression Scale: An updated literature review. *Journal of Psychosomatic Research, 52,* 69-77.

Buckner, L., & Yeandle, S. (2011). *Valuing Carers – Calculating the value of carers' support.* London: Carers UK.

Burton, C. M., & King, L. A. (2004). The health benefits of writing about intensely positive experiences. *Journal of Research in Personality, 38,* 150-163.

Burton, C. M., & King, L. A. (2008). Effects of (very) brief writing on health: The two-minute miracle. *British Journal of Health Psychology, 13,* 9-14.

Carers UK. (2007). *Real change not short change: Time to deliver for carers.* London: Carers UK.

Carers UK. (2008). *Carers in crisis*. London: Carers UK.

Frattaroli, J. (2006). Experimental disclosure and its moderators: A meta-analysis. *Psychological Bulletin, 132,* 823-865.

Haley, W. E., Roth, D. L., Howard, G., & Sarrord, M. M. (2010). Caregiving strain and estimated risk for stroke and coronary heart disease among spouse caregivers. *Stroke, 41*, 331-336.

Henry, J. D., & Crawford, J. R. (2005). The short form version of the Depression Anxiety Stress Scales (DASS-21): construct validity and normative data in a large non-clinical sample. *British Journal of Clinical Psychology, 44,* 227-239.

Hirst, M. (2005). Carer distress: A prospective, population-based based study. *Social Science & Medicine, 61,* 697-708.

Jones, C. J., Hayward, M., Brown, A., Clark, E., Bird, D., Harwood, G., Scott, C., Hillemann, A., & Smith, H. E. (2015). Feasibility and participant experience of a written emotional disclosure intervention for parental caregivers of people with psychosis. *Stress and Health.*

Lindon, D. (2007). *5 key facts about mental health carers*. London, EC; The Princess Royal Trust for Carers.

Luszczynska, A., Scholz, U., & Schwarzer, R. (2005). The general self-efficacy scale: multicultural validation studies. *The Journal of Psychology, 139,* 439-457.

Mackenzie, C. S., Wiprzycka, U. J., Hasher, L., & Goldstein, D. (2007). Does expressive writing reduce stress and improve health for family caregivers of older adults? *The Gerontologist, 47,* 296-306.

Mackenzie, C. S., Wiprzycka, U. J., Hasher, L., & Goldstein, D. (2008). Seeing the glass half full: Optimistic expressive writing improves mental health among chronically stressed caregivers. *British Journal of Health Psychology, 13,* 73-76.

Martinez-Rodriguez, S., Iraurgi, I., Gomez-Marroquin, I., Carraso, M., Ortiz-Marques, N., & Stevens, A. B. (2016). Psychometric properties of the leisure time satisfaction scale in family caregivers. *Psicothema, 28,* 207-213.

Martire, L. M., Lustig, A. P., Schulz, R., Miller, G. E., & Helgeson, V. S. (2004). Is it beneficial to involve a family member? A meta-analysis of psychosocial interventions for chronic illness. *Health Psychology, 23,* 599-611.

Martire, L. M., Manela, M., Shuttleworth, A., & Livingstone, G. (1997). An intervention study with husband and wife carers of older people with a psychiatric illness. *Journal of Affective Disorders, 46,* 279-284.

McHorney, C., Ware, J. E., Lu, R., & Sherbourne, C. D. (1994). The MOS 36-item short-form health survey (SF-36): III. Tests of data quality, scaling assumptions and reliability across diverse patient groups. *Medical Care, 32,* 40-66.

National Institute for Clinical Excellence (NICE). (2002). *Schizophrenia: core interventions in the treatment and management of schizophrenia in primary and secondary care.* London: NICE.

Oyebode, J. (2003). Assessment of carers' psychological needs. *Advances in Psychiatric Treatment, 9,* 45-53.

Parker, J. D. A., Taylor, G. J., & Bagby, M. R. (2003). The 20 item Toronto Alexithymia Scale III reliability and factorial validity in a community population. *Journal of Psychosomatic Research, 55,* 269-275.

Pinfold, V., & Corry, P. (2003). *Who Cares? The experience of mental health carers accessing services and information*. London: Rethink Mental Illness.

Pinquart, M., & Sorenson, S. (2003). Differences between caregivers and non-caregivers in psychological health and physical health: a meta-analysis. *Psychology & Ageing, 18,* 250-267.

Radcliffe, A. M., Lumley, M. A., Kendall, J., Stevenson, J. K., & Beltran, J. (2007). Written emotional disclosure: Testing whether social disclosure matters. *Journal of Social & Clinical Psychology, 26,* 362-384.

Royal College of General Practitioners. (2011). *Supporting Carers: An action guide for general practitioners and their teams* (2nd ed.). London, EC: The Princess Royal Trust for Carers.

Smith, H., Jones, C. J., Hankins, M., Field, A. P., Theadom, A., Bowskill, R., Horne, R., & Frew, A. (2015). The effects of expressive writing on lung function, quality of life, medication use and symptoms in adults with asthma: a randomised controlled trial. *Psychosomatic Medicine, 77,* 429-437.

Sorenson, S., Duberstein, P., Gill, D., & Pinquart, M. (2005). Dementia care: mental health effects, intervention strategies and clinical implications. *The Lancet Neurology, 5,* 961-973.

Tran, B. X., Ohinmaa, A., & Nguyen, L. T. (2012). Quality of life profile and psychometric properties of the EQ-5D-5L in HIV/AIDS patients. *Health and Quality of Life Outcomes, 10,* 132.
